# Numb and Numblike regulate sarcomere assembly and maintenance

**DOI:** 10.1172/JCI139420

**Published:** 2022-02-01

**Authors:** Baolei Wang, Min Yang, Shujuan Li

**Affiliations:** 1 West China Developmental & Stem Cell Biology Institute, West China Second University Hospital, Sichuan University, Chengdu, Sichuan, China.; 2 SARITEX Center for Stem Cell Engineering Translational Medicine, Shanghai East Hospital, Tongji University School of Medicine, Shanghai, China.; 3 Laboratory of Synthetic Embryology, Rockefeller University, New York, New York, USA.; 4 Children’s Hospital Affiliated to Zhengzhou University, Zhengzhou, Henan, China.

**Keywords:** Muscle Biology, Muscle

## Abstract

A sarcomere is the contractile unit of the myofibril in striated muscles such as cardiac and skeletal muscles. The assembly of sarcomeres depends on multiple molecules that serve as raw materials and participate in the assembly process. However, the mechanism of this critical assembly process remains largely unknown. Here, we found that the cell fate determinant Numb and its homolog Numblike regulated sarcomere assembly and maintenance in striated muscles. We discovered that Numb and Numblike are sarcomeric molecules that were gradually confined to the Z-disc during striated muscle development. Conditional knockout of *Numb* and *Numblike* severely compromised sarcomere assembly and its integrity and thus caused organelle dysfunction. Notably, we identified that Numb and Numblike served as sarcomeric α-Actin–binding proteins (ABPs) and shared a conserved domain that can bind to the barbed end of sarcomeric α-Actin. In vitro fluorometric α-Actin polymerization assay showed that Numb and Numblike also played a role in the sarcomeric α-Actin polymerization process. Last, we demonstrate that Numb and Numblike regulate sarcomeric α-Actinin–dependent (ACTN-dependent) Z-disc consolidation in the sarcomere assembly and maintenance. In summary, our studies show that Numb and its homolog Numblike regulate sarcomere assembly and maintenance in striated muscles, and demonstrate a molecular mechanism by which Numb/Numblike, sarcomeric α-Actin, and ACTN cooperate to control thin filament formation and Z-disc consolidation.

## Introduction

Numb, the cell fate determinant, regulates the asymmetric cell division of cortical neural progenitors in murine cortex and sensory organ precursors and neuroblasts in *Drosophila* (1–6). The function of Numb in different systems can be highly context dependent. Previous studies demonstrated that Numb and its homolog Numblike are required for myogenic stem cell maintenance and myoblast differentiation (7–9). During myogenesis, precursor myoblasts fuse with each other to form multinucleate myotubes, which triggers sarcomeric molecule expression and the subsequent assembly of the sarcomere. Although this process has been demonstrated, the molecular mechanism remains unclear. Numerous molecules related to cytoskeleton construction have been reported to be implicated in sarcomere assembly and maintenance (10–12). Previous studies have demonstrated that integrins pave the way for the initial steps of the myofibril assembly process (13–15), and that Numb controls integrin endocytosis for directional cell migration with atypical-protein kinase (αPKC) and its binding partner PAR-3 in HeLa cells (16), suggesting that Numb may play a role in sarcomere assembly. Numb and Numblike were proven to be essential for cardiac morphogenesis and progenitor differentiation and the loss of their functions resulted in defective sarcomeres in cardiomyocytes (CMs) (17, 18), which indicates a potential role for these 2 proteins in sarcomere formation and development. However, it is still unknown how Numb and Numblike are involved in the process of cardiomyocyte development. Here, we report that Numb and Numblike are sarcomeric α-Actin–binding proteins (ABPs) that localize to the Z-disc and regulate sarcomere assembly and maintenance in striated muscles. The functions of Numb and its homolog Numblike in regulating sarcomere assembly and maintenance indicate that Numb and Numblike may be targets for treating cardiomyopathy and myopathy.

## Results

### Numb and Numblike are required for sarcomere assembly and maintenance.

To better understand the functions of Numb and Numblike in heart development, we used *Mesp1-*cre to knock out the floxed alleles of *Numb* and *Numblike* (*Numb^fl/fl^* and *Numblike^fl/fl^*) in mouse cardiovascular precursors (Supplemental Figure 1A and Supplemental Table 1; supplemental material available online with this article; https://doi.org/10.1172/JCI139420DS1). Mesp1 is expressed in almost all precursors in the cardiovascular system. *Mesp1-cre* *Numb* and *Numblike* double-knockout (DKO) embryos were all aborted before embryonic day 8.5 (E8.5), but mice with solo *Numb* or *Numblike* knockout could live normally through adulthood (data not shown). Because *Mesp1-cre* DKO mice die at the very early developmental stage, it is difficult to observe the morphological defects in muscles caused by the lack of Numb or Numblike using this model.

We generated *MLC-2v-cre Numb* and/or *Numblike* knockout mice to investigate the loss of function of Numb and Numblike in early ventricular CMs. MLC-2v is a cardiac ventricular isoform of myosin light chain 2 and is initially expressed at about E8.0 (19). 5-Bromo-4-chloro-3-indolyl-β-D-galactoside (X-gal) staining verified that *MLC-2v-cre* was specifically expressed in the mouse heart ventricle at E9.5 (Supplemental Figure 1B). Immunofluorescence staining showed that in *MLC-2v-cre Numb* and *Numblike* DKO mice the Numb expression level sharply decreased in the heart ventricle at E10.5 (Supplemental Figure 1C). Due to the lack of availability in specific Numblike antibodies, RT-PCR was performed to detect *Numblike* expression. We found that *Numblike* transcription level was lower in the heart of *MLC-2v-cre* DKO mice at E10.5 (Supplemental Figure 1D). Because of the presence of other cell types in the heart tissue sample (e.g., superfluous fibroblasts and endothelial cells), the expression of *Numb* and *Numblike* was still detectable by immunofluorescence staining and RT-PCR. These *MLC-2v-cre* DKO mice could develop normally to adulthood without any apparent abnormality and therefore serve as an excellent model to probe if the absence of *Numb* and *Numblike* leads to any morphological defects during sarcomere formation.

**Figure 1:**
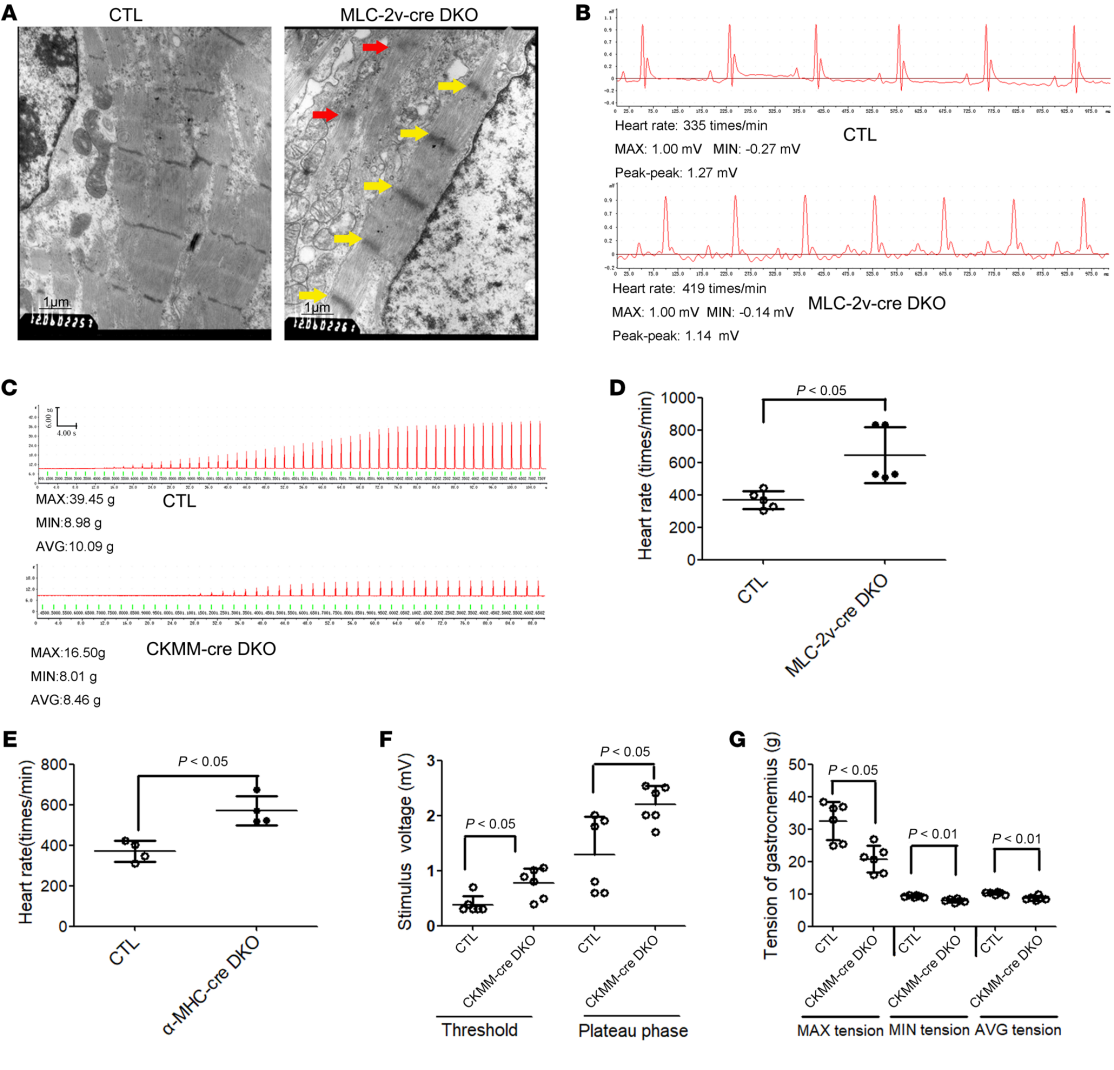
Numb and Numblike are required for sarcomere assembly. (**A**) Electron microscopy of the myocardial sarcomere of CTL and *MLC-2v-cre* DKO mice on E14.5 (original magnification, ×12,0000). (**B**) Electrocardiogram of CTL and *MLC-2v-cre* DKO mice on P60.5. (**C**) Electrophysiology of gastrocnemius of CTL and *CKMM-cre* DKO mice on P60.5. (**D**) Statistic data of heart rate of CTL and *MLC-2v-cre* DKO mouse on P60.5 (*n =* 5). (**E**) Statistic data of heart rate of CTL and α*-MHC-cre* DKO mice on P90.5 (*n =* 4). (**F**) Stimulus voltage (threshold and plateau) on gastrocnemius of CTL and *CKMM-cre* DKO mice on P60.5 (*n =* 6). (**G**) Maximum, minimum, and average tension of gastrocnemius of CTL and *CKMM-cre* DKO mice on P60.5 (*n =* 6). Scale bars: 1 μm. Two-tailed Student’s *t* test with error bars showing mean ± SD.

To investigate the changes of CM microstructure with Numb and Numblike mutations, we conducted transmission electron microscope (TEM) technology to the heart tissue of *MLC-2v-cre* DKO mice. Strikingly, broadened Z-disc (yellow arrows) and shortened sarcomere were observed in *MLC-2v-cre* DKO CMs at E14.5 (Figure 1A). Statistical data showed that the Z-disc width of the CMs in *MLC-2v-cre* DKO mice was 1.96 ± 0.99-fold broader than that of the CMs in the control group (CTL) WT mice, while the sarcomere length of the *MLC-2v-cre* DKO CMs was 0.8 ± 0.1-fold shorter than that of the CTL CMs. Surprisingly, the interval between the adjacent thin filaments was approximately 0.5 ± 0.01-fold narrower in the *MLC-2v-cre* DKO CMs than it was in the CTL CMs (Supplemental Table 2). Remarkably, many immature sarcomeres (red arrows) were also found in the *MLC-2v-cre* DKO CMs at E14.5 (Figure 1A). Additionally, mice with solo *Numb* or *Numblike* knockout with *MLC-2v-cre* exhibited normal sarcomeres (Supplemental Figure 1E and Supplemental Table 2). These results indicate that Numb and Numblike may play critical roles in sarcomere assembly and compensate each other in this process.

In addition to its assembly, the maintenance of sarcomeres is an important criterion for sarcomere function. Therefore, we investigated the role of Numb and Numblike in sarcomere maintenance by examining their function in postnatal sarcomeres. α*-*MHC is mainly expressed in the heart of adult mice (20, 21). To knock out Numb and Numblike in differentiated CMs, we crossed both *Numb^fl/fl^* and *Numblike ^fl/fl^* mice with α*-MHC-cre* mice. The results from Western blot analysis showed that the *Numb* expression level significantly decreased in the heart lysate of α*-MHC-cre* DKO mice at P60.5 (Supplemental Figure 1, F and G). RT-PCR results showed that the *Numblike* transcription level was lower in the heart of α*-MHC-cre* DKO mice (Supplemental Figure 2A). Similar to mice with the *MLC-2v-cre* DKO mutation, α*-MHC-cre* DKO mice could survive normally to adulthood without any abnormal behaviors (Supplemental Figure 2B). The myocardial sarcomere of α*-MHC-cre* DKO mice displayed an abnormal pattern similar to those of the *MLC-2v-cre* DKO mice (Supplemental Figure 2, C and D). The Z-disc width of the α*-MHC-cre* DKO CMs was 2.73 ± 0.85-fold broader than that of the CTL CMs, while the sarcomere length of the α*-MHC-cre* DKO CMs was 0.74 ± 0.07-fold shorter than that of the CTL CMs. Additionally, the interval between the adjacent thin filaments was approximately 0.48 ± 0.10-fold narrower in the α*-MHC-cre* DKO CMs than it was in the CTL CMs (Supplemental Table 2). Solo α*-MHC-cre* *Numb* or *Numblike* knockout mice exhibited normal sarcomere (data not shown).

To investigate the role of Numb and Numblike in both CMs and skeletal muscles (SKMs), another type of striated muscle, *CKMM-cre* transgenic mouse were used to knock out *Numb^fl/fl^* and *Numblike ^fl/fl^* in both CMs and SKMs. CKMM is highly expressed in mouse skeletal muscles and hearts. X-gal staining revealed that CKMM-cre was expressed in mouse hearts at E16.5 (Supplemental Figure 2E). Compared with the CTL mice, numb expression levels decreased in both CMs and SKMs of the *CKMM-cre* DKO mice, as determined by western blot analysis (Supplemental Figure 2, F–H). *Numblike* expression levels also decreased in CMs and SKMs of the *CKMM-cre* DKO mice, as indicated by RT-PCR (Supplemental Figure 2I). No significant behavioral defects were found in these *CKMM-cre* DKO mice (Supplemental Figure 2B). However, *CKMM-cre* DKO mice displayed a defective phenotype in the sarcomere similar to the phenotypes in *MLC-2v-cre* DKO and α*-MHC-cre* DKO mice (Supplemental Figure 3A). In SKMs, the Z-line width of the *CKMM-cre DKO* mice was 7.9 ± 2.75-fold broader than that of the CTL mice, while the sarcomere length of the *CKMM-cre DKO* mice was 0.69 ± 0.02-fold shorter than that of the CTL mice. The interval between adjacent thin filaments in the SKMs of *CKMM-cre* DKO mice was 0.5 ± 0.15-fold narrower than it was in the SKMs of CTL mice (Supplemental Table 2). In the CMs, the Z-disc width of the *CKMM-cre DKO* mice was 2.47 ± 1.03-fold broader than that of the CTL mice, while the sarcomere length of the *CKMM-cre DKO* mice was 0.85 ± 0.05-fold shorter than that of the CTL mice. The interval between adjacent thin filaments was approximately 0.51 ± 0.12-fold narrower in the *CKMM-cre* DKO CMs at P60 than it was in the CTL CMs (Supplemental Table 2). It is worth mentioning that solo *CKMM-cre* *Numb* or *Numblike* knockout mice exhibited normal sarcomeres (data not shown). Together, these results suggest that Numb and Numblike compensate each other and are critical in sarcomere maintenance.

Furthermore, to investigate whether the defective sarcomeres in CMs could trigger heart dysfunction, we used electrocardiogram analysis to assess the mouse heart function. The P-R interval, QRS complex, and S-T showed no significant difference between CTL and *MLC-2v-cre* DKO mice at P60.5 (data not shown), but the R-R interval was significantly shorter in the *MLC-2v-cre* DKO mice than that in the CTL mice. The P-R interval, the time from the onset of P wave to the start of QRS complex, reflects conduction through the AV node. QRS duration, the time interval from onset to the end of QRS complex, represents the depolarization of the ventricles. The ST segment, the interval between ventricular depolarization and repolarization, is identified as the end of the QRS complex to the beginning of the T wave. The mean values of R-R intervals in the CTL and the *MLC-2v-cre* DKO mice were 197.5 ± 9.95 ms (*n =* 5) and 206.67 ± 17.58 ms (*n =* 5), respectively (*P <* 0.05; Figure 1B). The average heart rate of *MLC-2v-cre* DKO mice was 645.2 ± 171.59 times/min (*n =* 5), which was significantly more rapid than the heart rate recorded for CTL mice at P60.5 (368.4 ± 55.67 times/min (*n =* 5; Figure 1C). α*-MHC-cre* DKO mice shared the same pattern in the electrocardiogram with *MLC-2v-cre* DKO mice (data not shown). Moreover, the heart rate of α*-MHC-cre* DKO mice was 570.75 ± 73.09 times/min (*n =* 6), which was significantly more rapid than the heart rate of CTL mice on P60.5 (the 369.75 ± 51.72 times/min (*n =* 6; Figure 1D).

We also sought to determine whether Numb and Numblike mutations lead to heart dilation, another important compensatory symptom for cardiac insufficiency. We detected various degrees of heart dilation in α*-MHC-cre* DKO mice (Supplemental Figure 3B). α*-MHC-cre* DKO mice has an approximately 2.1 ± 0.67-fold increase in heart volume compared with CTL mice on P60 (*n =* 4; Supplemental Figure 3C). Notably, *CKMM-cre* DKO mice had a more obvious dilation in the right ventricle (RV) compared with CTL mice (Supplemental Figure 3D).

In addition, the tension in the gastrocnemius muscle of *CKMM-cre* DKO mice was measured by electrophysiological equipment (Figure 1E). The stimulation voltage at the threshold and plateau were 0.78 ± 0.27 mV and 2.20±0.34 mV, respectively, in *CKMM-cre* DKO mice, which were significantly higher than the 0.38 ± 0.16 mV and 1.29 ± 0.69 mV obtained from CTL mice at P60 (Figure 1F). This finding indicates that the contraction sensitivity of skeletal muscles was affected by Numb and Numblike. Remarkably, the maximum, minimum, and average contraction tensions were 20.74 ± 4.15 g, 7.95 ± 0.53 g, and 8.73 ± 0.71 g, respectively, in *CKMM-cre* DKO mice, which were significantly lower than they were in CTL mice (32.52 ± 5.94 g, 9.3 ± 0.29 g, and 10.23 ± 0.41 g, respectively, Figure 1G). These data indicate a role of Numb and Numblike in the contractility of skeletal muscles. Together, these results suggest that knocking out *Numb* and *Numblike* led to defected sarcomeres, thus triggering organ dysfunction in striated muscles.

### Numb and Numblike are sarcomeric molecules that were gradually confined to Z-disc.

**Figure 2:**
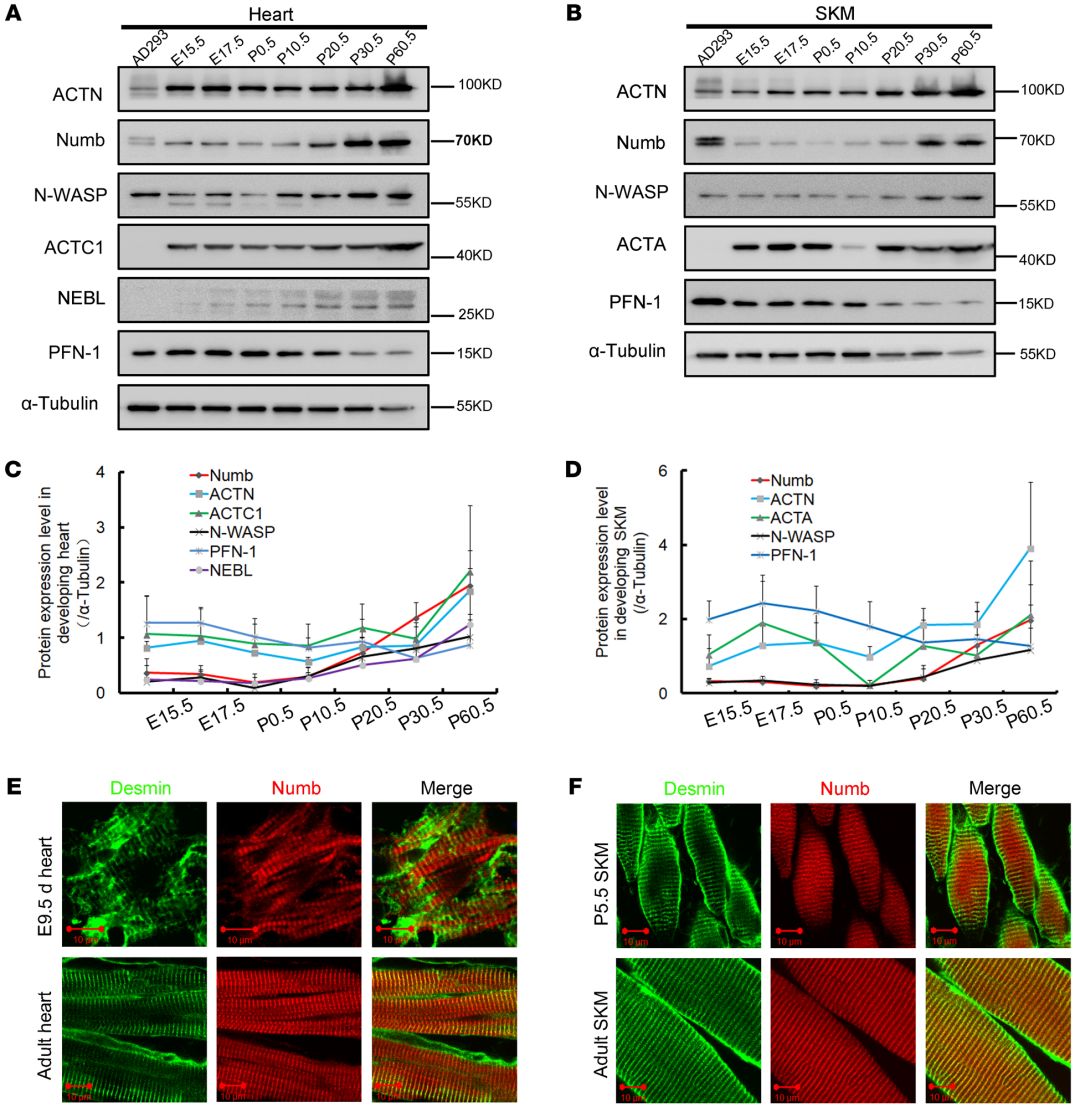
Numb is a sarcomeric molecule. (**A**) Western blots for Numb and other ABPs in the developing heart of WT (CTL) mice. (**B**) Western blots for Numb and other ABPs in the developing SKMs of CTL mice. (**C**) The protein expression level of Numb and other ABPs in the developing heart of CTL mice (*n =* 4) quantitated by densitometric analysis of Western blot. (**D**) The protein expression level of Numb and other ABPs in developing SKMs of CTL mice (*n =* 4) quantitated by densitometric analysis of Western blot. (**E**) Double immunofluorescence staining of Numb with Desmin in the CTL mouse heart on E9.5 and P60.5 (Desmin, green; Numb, red; original magnification, ×630, zoom in ×2). (**F**) Double immunofluorescence staining of Numb with Desmin in the mouse SKM on P5.5 and P60.5 (Desmin, green; Numb, red; original magnification, ×630, zoom in ×2). Data are mean ± SD. Scale bars: 10 μm.

To better understand the functions of Numb and Numblike in the striated muscle, we performed western blot to analyze the Numb expression dynamics during the development of mouse hearts and SKMs. From E15.5 to E17.5, the *Numb* expression level remained stable in both types of striated muscle and then significantly decreased from P0.5 to P10.5, followed by a sharp increase after P10.5. Meanwhile, we also examined the expression level of several muscle-specific Actins (ACTC1 for cardiac muscle and ACTA for skeletal striated muscle) and ABPs (α-Actinin [ACTN], Neural Wiskott-Aldrich syndrome protein 1 [N-WASP], Nebulette [NEBL], and Profilin-1 [PFN-1]). The observed Numb expression trend coincided with the expression of ACTC1/ACTA and ACTN in both heart and skeleton muscles (Figure 2, A–D). To eliminate the potential interference from superfluous fibroblasts and/or endothelial cells in the tissue samples, we confirmed the Numb expression in the mouse SKM myoblast cell line C2C12 and the rat cardiac myoblast cell line H9C2 by Western blot (Supplemental Figure 3, E and F). Notably, the Numb expression trend was similar to that of ACTC1/ACTA and ACTN in both undifferentiated and differentiated C2C12 cells (Supplemental Figure 3, F and G). In addition, we compared the Numb expression level between the atrium and ventricle by immunofluorescence staining on E9.5 and Western blot on P60.5. Interestingly, the Numb expression level was higher in the ventricle than it was in the atrium, aligning with that of the ACTC1 and ABPs (Supplemental Figure 4, A–C).

To identify the localization of Numb in striated muscles, double immunofluorescence staining of Numb (rabbit anti-Numb antibody) and Desmin (mouse anti-Desmin antibody) was performed in the hearts of CTL mice on E9.5 and P60.5. Desmin is an intermediate filament protein localized at the Z-disc in striated muscles. Surprisingly, we found that Numb was gradually confined to the Z-disc in the longitudinal section of the CMs (Figure 2E). We next sought to investigate whether Numb has the same expression pattern in SKMs as it does in CMs. We performed the same double immunofluorescence staining on the gastrocnemius muscles in CTL mice on P5.5 and P60.5, and found that Numb was also gradually confined to the Z-disc in the longitudinal sections of the SKMs (Figure 2F). Additionally, we detected a dendritic Numb expression pattern of the Z-disc in the transection of the CMs (Supplemental Figure 4D). To sufficiently validate our new findings, we also stained Numb in the H9C2 cells. We found that Numb granules aligned to form a sarcomeric pattern after 48 hours in culture (Supplemental Figure 4E). We also evaluated the specificity of the antibody by comparing different Numb antibodies manufactured by different suppliers and detected the same sarcomeric expression pattern of Numb in striated muscles (Supplemental Figure 4F). These results indicate that Numb is present in striated muscles in a location-specific matter.

Vertebrates express different isoforms of Numb and Numblike, and presumably these isoforms have different functions (Supplemental Figure 4G). A previous study showed that both short and long isoforms of Numb are expressed in human embryonic kidney 293 (HEK293) cells (22). However, we found that only the short *Numb* isoforms were expressed in CMs and SKMs (Figure 2, A and B). In order to avoid interference from other cell types in the tissue, we also analyzed the Numb isoforms in H9C2 and C2C12 cells. We found that only 2 short Numb isoforms (Numb65 and Numb66) were present in these cells (Supplemental Figure 4, H and I). To eliminate the possibility of false negatives due to Numb protein modification, we conducted RT-PCR with primers that can distinguish the 2 short Numb isoforms. The length difference of the target sequence of the 2 isoforms is 33 bp, attributed to the phosphotyrosine-binding domain (PTB) insert sequence. The PTB domain that contains the insert sequence is abbreviated as PTB_L_, and the PTB domain without the insert sequence is abbreviated as PTB_S_. Consistent with the Western blot result (Supplemental Figure 4I), both PTB_L_ and PTB_S_ were detected in the C2C12 cells (Supplemental Figure 4J) by RT-PCR. Notably, both RT-PCR and Western blot showed that the expression level of PTBs was higher than that of PTB_L_ in the H9C2 and C2C12 cell lines (Supplemental Figure 4, H–J). On the other hand, the RT-PCR result showed that both Numb and Numblike were expressed in the H9C2 and C2C12 cell lines (Supplemental Figure 4K). Together, these results indicated that two Numb short isoforms (Numb65 and Numb66) and Numblike were coexpressed in the studied striated muscles (CMs and SKMs). To confirm these findings, we also cloned the cDNA of the 4 isoforms of *Numb* (Numb65, Numb66, Numb71, and Numb72) and *Numblike* with the enhanced green fluorescence protein (EGFP) tagged at the N-terminus. The corresponding constructed plasmids were transfected into the SKMs of the living mouse using an in vivo electroporation technique. Four days after electroporation, the SKMs were surgically removed and examined for the fluorescence expression of the Numb isoforms and Numblike by confocal microscopy (Supplemental Figure 5A). Interestingly, the Numb isoforms and Numblike had different expression patterns in the longitudinal sections of the SKMs. Specifically, the short isoforms of Numb (Numb65 and Numb66) and Numblike were aligned and formed a regular nemaline appearance (Supplemental Figure 5B), while the long isoforms of Numb (Numb71 and Numb72) concentrated to form large particles. These results indicate that short isoforms of Numb and Numblike are present in striated muscles and may function differently in sarcomeres.

### Numb and Numblike are sarcomeric ABPs.

**Figure 3:**
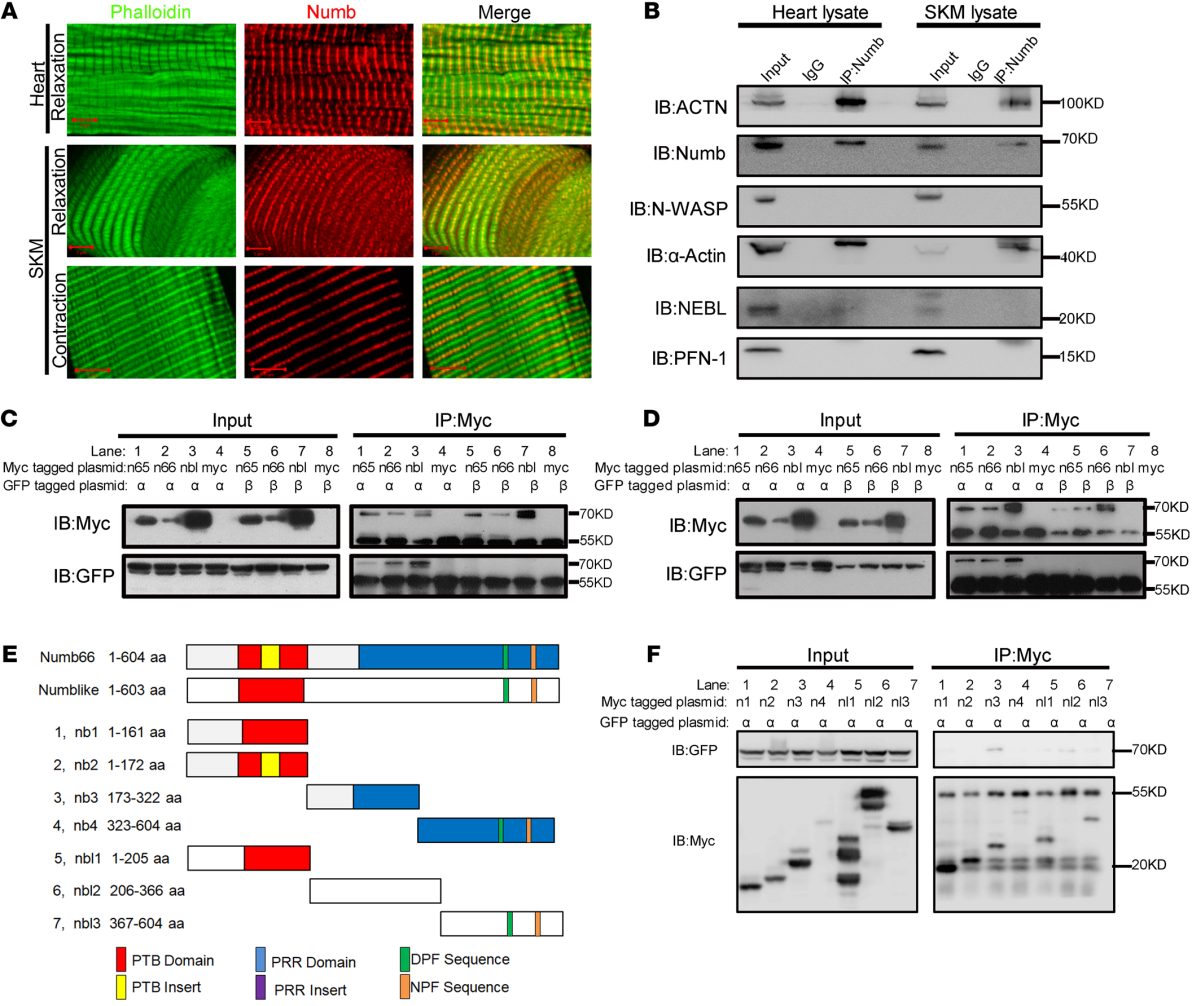
Numb and Numblike interact with sarcomeric α-Actin. (**A**) Double immunofluorescence staining of Numb with phalloidin in CMs and SKMs of mice on P60.5 (phalloidin, green; Numb, red; original magnification, ×630, zoom in ×4). (**B**) Coimmunoprecipitation of Numb with other sarcomeric ABPs in heart and SKM lysates. (**C**) Coimmunoprecipitation of the transfected Myc-Numb65, Myc-Numb66, Myc-Numblike with EGFP–α-Actin (ACTC1) and EGFP–β-Actin in AD293 cells. (**D**) Coimmunoprecipitation of the transfected Myc-Numb65, Myc-Numb66, and Myc-Numblike with EGFP–α-Actin (ACTA) and EGFP–β-actin in the conducted AD293 cells. (**E**) Schematic of Myc-*Numb* and Myc-*Numblike* truncation. (**F**) Coimmunoprecipitation of the transfected truncated Myc-Numb and Myc-Numblike with EGFP–α-Actin (ACTC1) in AD293 cells. Scale bars: 5 μm.

To investigate the molecular mechanisms through which Numb and Numblike regulate sarcomere assembly and maintenance, we performed double immunofluorescence staining of Numb and other sarcomeric molecules, including Actins and several ABPs in isolated CMs (Supplemental Figure 5, C–E). We found that Numb colocalized with α-Actin/phalloidin at the barbed end of the thin filament in both CMs and SKMs (Figure 3A). The double immunofluorescence staining of Numb and α-Actin/phalloidin in H9C2 cells after 2 hours and 48 hours in culture showed that Numb is present with Actin filaments (Supplemental Figure 6, A and B). To identify whether Numb and Numblike are sarcomeric ABPs, we coimmunoprecipitated Numb with muscle-specific α-Actin (ACTC1/ACTA) and several sarcomeric ABPs (ACTN, N-WASP, NEBL, and PFN-1) from the heart and the SKM lysates of mice. We found that Numb immunoprecipitated with sarcomeric Actin (ACTC1/ACTA) and ACTN (Figure 3B).

Next, we sought to investigate whether the 2 short isoforms of Numb (Numb65 and Numb66) and Numblike interact with sarcomeric α-Actin (ACTC1/ACTA) and ACTN. Plasmids with the red fluorescence protein (RFP) tagged *Numb65*, *Numb66*, and *Numblike* cDNA were constructed and then cotransfected, respectively, with EGFP-tagged *ACTC1/ACTA* into AD293 cells. AD293 is a cell line derived from the standard HEK293 cells, with improved cell adherence and plaque formation properties. We chose the AD293 cell line for this experiment because of its superior transfection trait, attributed to the large surface area of the cells. To determine whether Numb and Numblike specifically interact with striated muscle-specific Actin, we cotransfected Numb and Numblike with EGFP-tagged β-Actin to AD293 cells as the comparison. β-Actin is a nonmuscle cytoskeletal Actin. We observed that Numb65, Numb66, and Numblike colocalized with the ACTC1/ACTA aggregates 24 hours after the cotransfection, while no colocalization of Numb65, Numb66, or Numblike with fibroid β-Actin was detected (Supplemental Figure 7, A–C). Additionally, ACTC1/ACTA aggregated to form clusters after we transfected the EGFP- *ACTC1/ACTA* plasmid alone into the AD293 cells (Supplemental Figure 8A), while Numb65/Numb66/Numblike, transfected alone, was diffused in the cells 24 hours after the transfection (Supplemental Figure 8B). ACTN2 and ACTN3 are sarcomeric ACTNs highly expressed at the Z-disk of the striated muscle. We failed to detect the colocalization of Numb65, Numb66, or Numblike with ACTN2 (Supplemental Figure 8C). For coimmunoprecipitation confirmation, plasmids with Myc-tagged *Numb65*, *Numb66*, and *Numblike* were constructed and cotransfected with plasmids with EGFP-tagged *ACTC1/ACTA* (or the control EGFP-tagged β*-Actin*) into AD293 cells. Numb65, Numb66, and Numblike immunoprecipitated with ACTC1/ACTA but not β-Actin 24 hours after the cotransfection (Figure 3, C and D). Also, we failed to detect interactions between the Numb65, Numb66, or Numblike and ACTN2/ACTN3 in the coimmunoprecipitation experiment (Supplemental Figure 8, D and E), suggesting an indirect interaction between Numb/Numblike and sarcomeric ACTN in vivo. Together, these results suggest that Numb and Numblike are ABPs that specifically bind to α-Actins that are expressed in striated muscles.

Numb and Numblike share the PTB domain at their N-terminus and the proline-rich region (PRR) domain at the C-terminus (Supplemental Figure 4F). To investigate which domains in Numb and Numblike bind to sarcomeric α-Actin, we constructed Myc-tagged *Numb* and *Numblike* plasmids with different lengths of amino acid residues (Figure 3E), which were then cotransfected with EGFP-tagged *ACTC1* plasmid into AD293 cells, respectively. The cells were coimmunoprecipitated 24 hours after transfection to analyze the protein interaction. The results indicate that Numb (173–322 aa) and Numblike (206–366 aa) were the dominant residue sequences that interacted with ACTC1 and that Numblike (1–205 aa) and Numblike (367–604 aa) had negligible functional interactions with ACTC1 (Figure 3F). After blasting Numb (173–322 aa) and Numblike (206–366 aa) amino acid sequences in the NCBI database, we found that these 2 sequences were highly conserved, indicating the potentially critical role of these conservative domains in α-Actin binding (Supplemental Figure 9A).

Using cotransfection in AD293 cells, we also examined the interactions between Numb65, Numb66, and Numblike and other EGFP-tagged PRR domain-interaction proteins, including *PFN-1*, *NEB*-SH3 domain, *NEBL, NEBL*-SH3 domain, and the *N-WASP*-EVH1 domain. We did not detect any interactions of these domains with Numb and Numblike (Supplemental Figure 9, B–G).

### Numb and Numblike interact with the barbed end domain of α-Actin.

Actin is a globular protein that has one large and one small domain. The small domain consists of subdomain 1 (residues 1–32, 70–144, and 338–375) and subdomain 2 (residues 33–69) and the large one consists of subdomain 3 (residues 145–180 and 270–337) and subdomain 4 (residues 181–269) (23). To determine which domains in α-Actin interact with Numb and Numblike, we constructed plasmids with EGFP-tagged *ACTC1* residue sequences (Supplemental Figure 10A) and then cotransfected them with Myc-tagged *Numb65*, *Numb66*, and *Numblike* into AD293 cells. The coimmunoprecipitation results showed that Numb (Numb65 and Numb66) and Numblike interacted with ACTC1 (78–163 aa) and ACTC1 (262–377 aa) (Supplemental Figure 10, B and C). To investigate if some specific residues are crucial for ACTC1 to form an aggregated pattern, we transfected the 4 EGFP-tagged *ACTC1* residue sequences alone into AD293 cells, respectively. We found that ACTC1 (78–163 aa) and ACTC1 (262–377 aa) aggregated 24 hours after the transfection while the other 2 residue sequences, ACTC1 (1–77 aa) and ACTC1 (164–261 aa), were dispersed in the AD293 cells (Supplemental Figure 10D). For further validation, we also cotransfected these sequences with RFP-*Numb65*, RFP-*Numb66*, and RFP-*Numblike*. The same aggregated patterns were found in cells transfected with ACTC1 (78–163 aa) and ACTC1 (262–377 aa) colocalizing with Numb and Numblike (data not shown).

Furthermore, by comparing the amino acid sequences of murine muscle-specific α-Actin (ACTC1/ACTA) to β-Actin, we identified 2 additional amino acids in ACTC1/ACTA. We also identified a total of 24 amino acids in ACTC1/ACTA that differ from their counterparts in β-Actin. Among these 24 amino acids, 4 amino acids (I78, T105, V131, and L155) were in *ACTC1* (78–163 aa), and 9 amino acids (T262, I269, A274, Y281, I289, N299, M301, T360, and A367) were in *ACTC1* (262–377 aa, Supplemental Figure 11A). These amino acids might be critical for the interactions of α-Actin with Numb and Numblike and contribute to the binding specificity of Numb and Numblike to sarcomeric α-Actin.

### Numb and Numblike regulate sarcomeric α-Actin filament formation.

We demonstrated that Numb and Numblike are sarcomeric ABPs. However, how they function upon binding to sarcomeric α-Actin is unknown. The expression level of sarcomeric α-Actin was not changed by the conditional knockout of *Numb* and *Numblike* in mice (Supplemental Figure 2, F–H). The double knockdown of *Numb* and *Numblike* in the H9C2 cell line also did not result in changes in sarcomeric α*-Actin* expression (Supplemental Figure 11, B–D). However, the *Numb* expression level was remarkably decreased by the *ACTC1*-knockdown in the H9C2 cell line (Supplemental Figure 11, E and F), suggesting that sarcomeric *ACTC1* contributes to the regulation of Numb expression in striated muscles.

**Figure 4:**
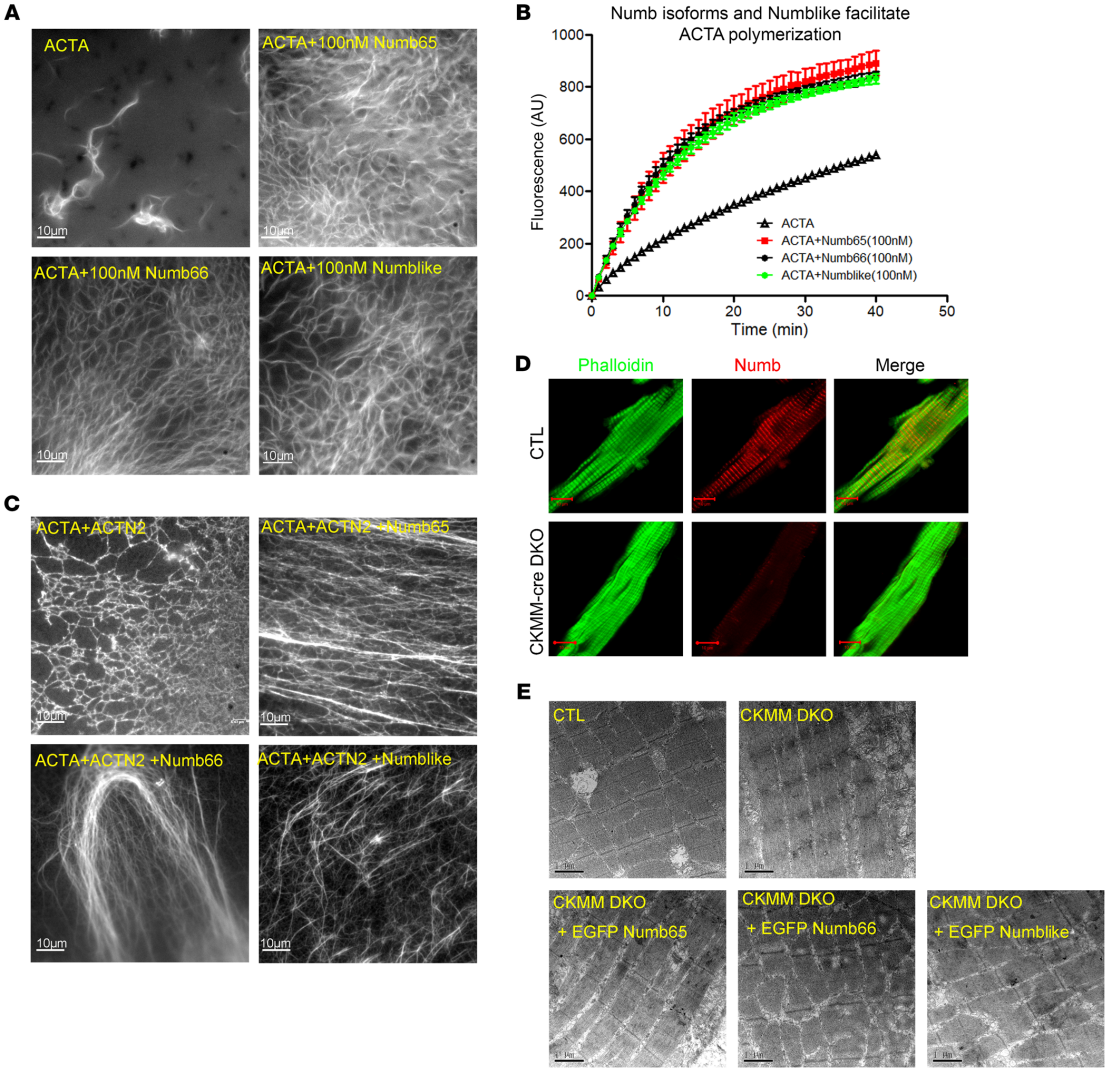
Numb and Numblike regulate sarcomeric α-Actin filament formation. (**A**) TIRFM micrographs of Actin (ACTA) filaments 10 minutes after initiating polymerization of Mg-ATP-Actin (1 mM) with 100 nM His-Numb65, His-Numb66, and His-Numblike (original magnification, ×1000). (**B**) Time course of fluorescence increase upon polymerization of 2 mM Mg-ATP-Actin (6% pyrene-labeled) with 100 nM His-Numb65, His-Numb66, and His-Numblike (*n =* 3). Data are mean ± SD. (**C**) TIRF micrographs of Actin (ACTA) filaments 10 minutes after initiating polymerization of 1 mM Mg-ATP-Actin with 100 nM *A*CTN2 and 100 nM His-Numb65, His-Numb66, and His-Numblike, respectively (original magnification, ×1000). (**D**) Double immunofluorescence staining of Numb and phalloidin in the SKMs of CTL and *CKMM-cre* DKO mice on P60.5 (phalloidin, green; Numb, red; original magnification, ×630, zoom in ×2). (**E**) Electron microscopy of the gastrocnemius muscle of *CKMM-cre* DKO mice electroporated with EGFP-tagged *Numb65*, *Numb66*, and *Numblike* (original magnification, ×20,000). Scale bars: 10 μm (**A**, **C**, **D**) or 1 μm (**E**).

Knocking out *Numb* and *Numblike* not only broadened the Z-disc width but also shortened the sarcomere length. Sarcomere length is represented by the length of its thin filaments to a great extent. Since Numb and Numblike are located at the barbed end of the thin filament, which is the α-Actin nucleation and polymerization site, we speculate that Numb and Numblike may have a role in sarcomeric thin filament formation. To test this hypothesis, we obtained the purified Numb and Numblike proteins from the AD293 cells transfected with His-tagged *Numb*65, *Numb*66, and *Numblike* and investigated if and how these proteins affect the Actin polymerization (Supplemental Figure 12, A and B). Actin polymerization can be reflected and measured by the emitted intensity (at 386 nm) of pyrene since it increases markedly upon polymerization, and this increased intensity is insensitive to filament quantity and length. Surprisingly, using the total internal reflection fluorescence microscope (TIRFM), we found that Numb and Numblike facilitated α-Actin polymerization (Figure 4A). Meanwhile, the pyrene-labeled ACTA omitted higher fluorescence intensity with the presence of 100 nM Numb65, Numb66, and Numblike (Figure 4B). Numb65, Numb66, and Numblike also elongated unbranched α-Actin filament formation in the presence of ACTN2 (Figure 4C). We also found that applying α-Actin polymerization inhibitors (cytochalasin B, cytochalasin D, and latrunculin A) to the H9C2 cells failed to disrupt the colocalization of Numb with ACTC1 (Supplemental Figure 13, A–D). The expression levels of Numb and the other APBs remained unaffected by the 24-hour treatment of the cytochalasins or latrunculin (Supplemental Figure 12C), indicating that the binding site of α-Actin to Numb was not blocked by α*-Actin* polymerization inhibitors.

### Overexpressed Numb and Numblike prolong thin filaments in vivo.

We found that Numb and Numblike facilitated α-Actin polymerization and knocking out *Numb* and *Numblike* impaired the thin filaments in sarcomeres (Figure 4D). Therefore, we hypothesized that the overexpression of Numb and/or Numblike could elongate the thin filament of CTL mice in vivo. To test this hypothesis, we overexpressed EGFP-tagged *Numb*65, *Numb*66, and *Numblike*, respectively, in CTL gastrocnemius muscle cells by in vivo electroporation. Four days after the electroporation, we stained the cells with fluor-555 phalloidin, and found that the overexpression of the 2 *Numb* isoforms and *Numblike* prolonged thin filaments (Supplemental Figure 12D).

Next, to investigate if the defective sarcomeres of DKO mice could be rescued by overexpressing *Numb* isoforms and *Numblike*, we respectively overexpressed EGFP-tagged *Numb*65, *Numb*66, and *Numblike* in CKMM DKO gastrocnemius muscle by in vivo electroporation. TEM was conducted 4 days after the electroporation. Strikingly, defective sarcomeres of DKO mice were partially rescued by the overexpression of 2 short isoforms of Numb and Numblike (Figure 4E).

### Numb and Numblike regulate Z-disc assembly and integrity through ACTN.

**Figure 5:**
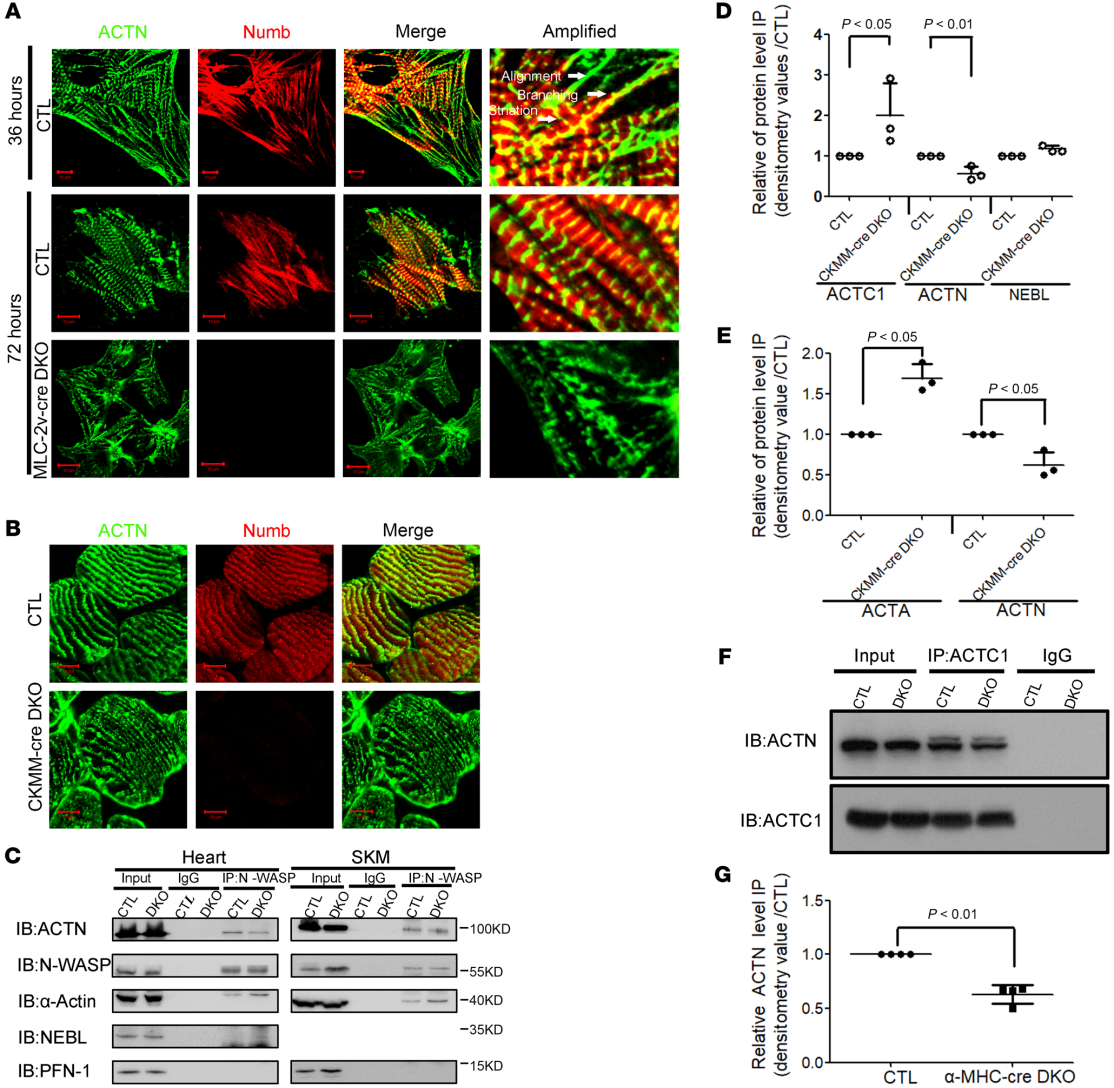
Numb and Numblike regulate Z-disc assembly and integrity through *ACTN*. (**A**) Double immunofluorescence staining of Numb and ACTN in the CMs of E14.5 CTL and *MLC-2v-cre* DKO mice after 72-hour culture in vitro (ACTN, green; Numb, red; original magnification, ×630, zoom in ×2; amplified images: original magnification, ×630, zoom in ×5). (**B**) Double immunofluorescence staining of Numb and ACTN in the SKMs of *CKMM-cre* DKO mice on P60 (ACTN, green; Numb, red; original magnification, ×630, zoom in ×2). (**C**) Coimmunoprecipitation of N-WASP with other ABPs in heart and SKM lysates of CTL and *CKMM-cre* DKO mice on P60. (**D** and **E**) Relative protein level of ABPs immunoprecipitated by N-WASP in heart and SKM lysates of CTL and *CKMM-cre* DKO mice on P60 (*n =* 3). (**F**) Coimmunoprecipitation of α-Actin (ACTC1) with ACTN in heart lysates of CTL and α*-MHC-cre DKO* mice on P60. (**G**) Relative ACTN protein level immunoprecipitated by ACTC1 in heart lysates of CTL and α*-MHC-Cre* DKO mice on P60 (*n =* 4). Scale bars: 5 μm. Two-tailed Student’s *t* test with error bars showing mean ± SD.

The integrity and stability of Z-disc are vital for sarcomere functions. Sarcomeric ACTN, an important marker for Z-disc assembly and integrity, is localized at the Z-disc and cross-links in the thin filaments in striated muscles. Numb and Numblike immunoprecipitated with ACTN in both heart and SKM lysates, albeit the interactions were indirect. Here, using an in vitro CM differentiation assay, we aimed to investigate whether Numb and Numblike function to sustain ACTN integrity. CMs were isolated from CTL mouse hearts on E14.5 and then cultured for 36 hours in vitro. Three types of ACTN morphology (alignment, branching, and striation) were detected after the differentiation (Figure 5A), which could represent 3 successive phases of Z-disc assembly in sarcomeres. Surprisingly, Numb was found localized to all 3 types of ACTN, indicating a potential role of Numb in the Z-disc assembly process. When CMs were cultured 72 hours in vitro, the aligned and branched ACTN can hardly be identified, and, therefore, the integrated Z-disc is reflected by striated ACTN. The striated-associated ACTN seems largely distraught in the *MLC-2v-cre* DKO CMs that were cultured 72 hours after isolation (Figure 5A). This intermittent ACTN phenotype features the defective Z-disc caused by Numb and Numblike mutations and suggests that Numb and Numblike regulated Z-disc assembly through the mediation of ACTN. To investigate if Numb and Numblike maintain Z-disc integrity with ACTN, we performed double immunofluorescence staining of Numb and ACTN in the gastrocnemius muscle of CTL and *CKMM-cre* DKO mice on P60. The tissue from *CKMM-cre* DKO mice demonstrated a defective ACTN (Figure 5B), suggesting that Z-disc integrity might be regulated by Numb and Numblike through mediating ACTN.

To investigate whether Numb and Numblike maintain the interaction between α-Actin and ACTN in the Z-disc, we conducted coimmunoprecipitation using N*-*WASP antibody in both the heart and SKM lysates from CTL and *CKMM-cre* DKO mice on P10.5. We found that this ABP could recruit more sarcomeric α-Actin but less sarcomeric ACTN in both the CMs and SKMs from *CKMM-cre* DKO mice compared with the control (Figure 5, C–E). Therefore, we speculate that Numb and Numblike may impact the interactions between sarcomeric α-Actin and sarcomeric ACTN. Coimmunoprecipitation results of heart lysate obtained on P10.5 using α-Actin antibody also confirmed that sarcomeric ACTC1 recruited fewer ACTN in the CMs of α*-MHC-cre* DKO mice (Figure 5F). The statistical data showed that the interaction between sarcomeric ACTC1 and ACTN decreased by 0.63-fold in the CMs of α*-MHC-cre* DKO mice compared with this interaction in the CMs of CTL mice (Figure 5G). Together, these results indicate that Numb and Numblike regulate sarcomere assembly and integrity by maintaining the interaction between α-Actin and ACTN.

### Numb and Numblike cooperate with sarcomeric ACTN in maintaining thin filament morphology.

Since Numb and Numblike impact the interactions between sarcomeric α-Actin and ACTN, we therefore speculated that Numb and Numblike may also regulate the thin filament morphology by cooperating with ACTN. To verify this speculation, we conducted the TIRFM to investigate if the presence of purified Numb and Numblike affect the actin polymerization of ACTA together with ACTN2. Strikingly, Numb65, Numb66, and Numblike cooperated with ACTN2 and ACTA to form rectilinear actin filament, while the formed actin filament appeared to be reticular without Numb and Numblike (Figure 4C). These data indicate that short Numb isoforms and Numblike are critical for ACTN2-dependent thin filament formation.

## Discussion

Our study identified the role of Numb and its homolog Numblike in regulating sarcomere assembly and maintenance in striated muscles of both embryonic and adult mice. The results from this work revealed that Numb and Numblike served as sarcomeric α-Actin–binding proteins and corporate with sarcomeric ACTN to regulate Z-disc consolidation and contribute to the assembly and maintenance of sarcomere. It is already known that Numb family proteins are essential for cardiac morphogenesis and progenitor differentiation (18). In mice, Numb has been reported to express in adult cardiac progenitor cells and be involved in progenitor asymmetric division (24, 25). Previous studies have also shown that Numb and Numblike are essential for the regulation of myocardial compaction (17). However, the underlying mechanism of how Numb and Numblike participate in cardiac development remains unknown. Our discovery of the critical role that Numb and Numblike played in the sarcomere of striated muscle tissues shed some new light on how these proteins are involved in and regulate heart function.

In this study, we generated several lines of conditional *Numb* and/or *Numblike* KO mice to investigate the effect of these 2 proteins on the development and maintenance of the heart at different stages, and to inspect how they affect the sarcomere formation in striated muscles. We first generated Mesp1-cre DKO mice. However, since Mesp1 is expressed in the mesoderm component of the amnion and almost all precursors of the cardiovascular system, the loss of function of Numb and Numblike with Mesp1 expression is fatal to the mice at the very early developmental stage. In order to investigate the role of Numb and Numblike specifically in early cardiac development, we generated *MLC-2v-cre* DKO mice. Since the expression of MHC-2v is restricted within the ventricular chamber throughout the mouse embryonic development, this mouse line serves as a great platform to study how Numb and Numblike affect sarcomere assembly in ventricular CMs at the early stage (19, 26). We also generated α*-MHC-cre* DKO mice to investigate the functions of Numb and Numblike in sarcomere maintenance at the later stage. α-MHC is temporarily expressed at a rather low level at the early embryonic stage (E7–11) and dominantly expressed after birth and in the heart of adult mice at a very high level (20, 22). Therefore, α*-MHC-cre* DKO mice serve as a good model to probe how Numb and Numblike affect the maintenance of already-assembled sarcomeres in mouse hearts. Last, we generated *CKMM-cre* DKO to investigate the role of Numb and Numblike in CMs and SKMs since CKMM is highly expressed as a structural protein in both these 2 types of striated muscle (27, 28). From the phenotype results of these mouse lines, we verified that Numb and Numblike not only regulate sarcomere assembly but also contribute to the maintenance of the sarcomere structure. Using these mouse models, we observed that the loss of function of Numb and Numblike, regardless of the stage, led to heart dysfunction, including rapid heart rate and dilated heart. Additionally, we also identified that the mutation of Numb and Numblike affects the contractility ability of skeletal muscles. These findings hold great clinical significance, as they pinpointed additional potential causes for cardiomyopathy and myopathy under developmental and pathological conditions such as dilated cardiomyopathy and myasthenia. The knowledge provided in this study offers new potential implications for future clinic therapy.

Our data demonstrated that Numb and Numblike are sarcomeric ABPs that are localized at the barbed end of the thin filament in the Z-disc, and which contribute to the understanding of the underlying mechanism of sarcomeric thin filament formation and Z-disc integrity. Numb and Numblike have been demonstrated to play crucial roles in stem cell fate determination (1), asymmetric cell division (2–4), endocytosis (29), cell adhesion (30), and cell migration (31). However, if and how Numb and Numblike function in striated muscles remain unelaborated. In muscle tissues, sarcomeric actin filaments serve as the most dynamic organelle and undergo multiple and complex assembly mechanisms to meet the needs of organismal activity. α-Actin is the principal component of the thin filament and a major constituent of the contractile apparatus in the muscle tissue. α-Actin functions through binding with ABPs in the formation of sarcomeric α-Actin filament. To date, a series of ABPs have been identified to function together with α-Actin in this formation process. For example, Nebulin and N-WASP form a complex and contribute to Actin nucleation for unbranched Actin filament formation from the Z bands (32). Leiomodin has been identified as the filament nucleator located in the pointed end of the thin filament and is involved in the nucleation mechanism of Actin (33). Fhod3, located near the middle of the sarcomere, regulates the elongation rate of the barbed end and may protect the barbed end for Actin assembly in sarcomere formation (34). Our findings classified Numb and Numblike into this sarcomeric ABP category and shed new light on the underlying mechanism of the formation of striated muscle sarcomeres.

Moreover, we detected highly conserved regions of amino residues shared by Numb and Numblike that bind to the barbed end domain of α-Actin, providing intrinsic information for discovering other potential sarcomeric ABPs in the future. Numb and Numblike bind to ACTC1 in CMs and ACTA in SKMs. Therefore, we speculate that the interaction between Numb/Numblike and α-Actin is universally conserved, and the other Actins, such as smooth muscle α-Actin (α-SMA) that shares a highly conserved amino residue with ACTC1/ACTA, may also interact with Numb and Numblike. Numb and Numblike may also play unique roles in cells that contain other α-Actins.

In total, 6 Numb isoforms, each with different functions, were found in humans. Specifically, the Numb55 and Numb56 isoforms, identified according to their molecular weight, are distinctively expressed in cancer cells (35). The other 4 isoforms of Numb—Numb65, Numb66, Numb71, and Numb72—and Numblike are ubiquitously expressed in all tissues. Numb65 and Numb66 are often identified as the short isoforms of Numb, while Numb71 and Numb72 are identified as the long isoforms. Our results show that only the short isoforms of Numb were expressed in striated muscles, suggesting that the functions of Numb isoforms are highly context-dependent. These short isoforms of Numb and Numblike cooperate with sarcomeric ACTN in maintaining thin filament morphology in terms of supporting Z-disc assembly and maintaining its integrity.

**Figure 6:**
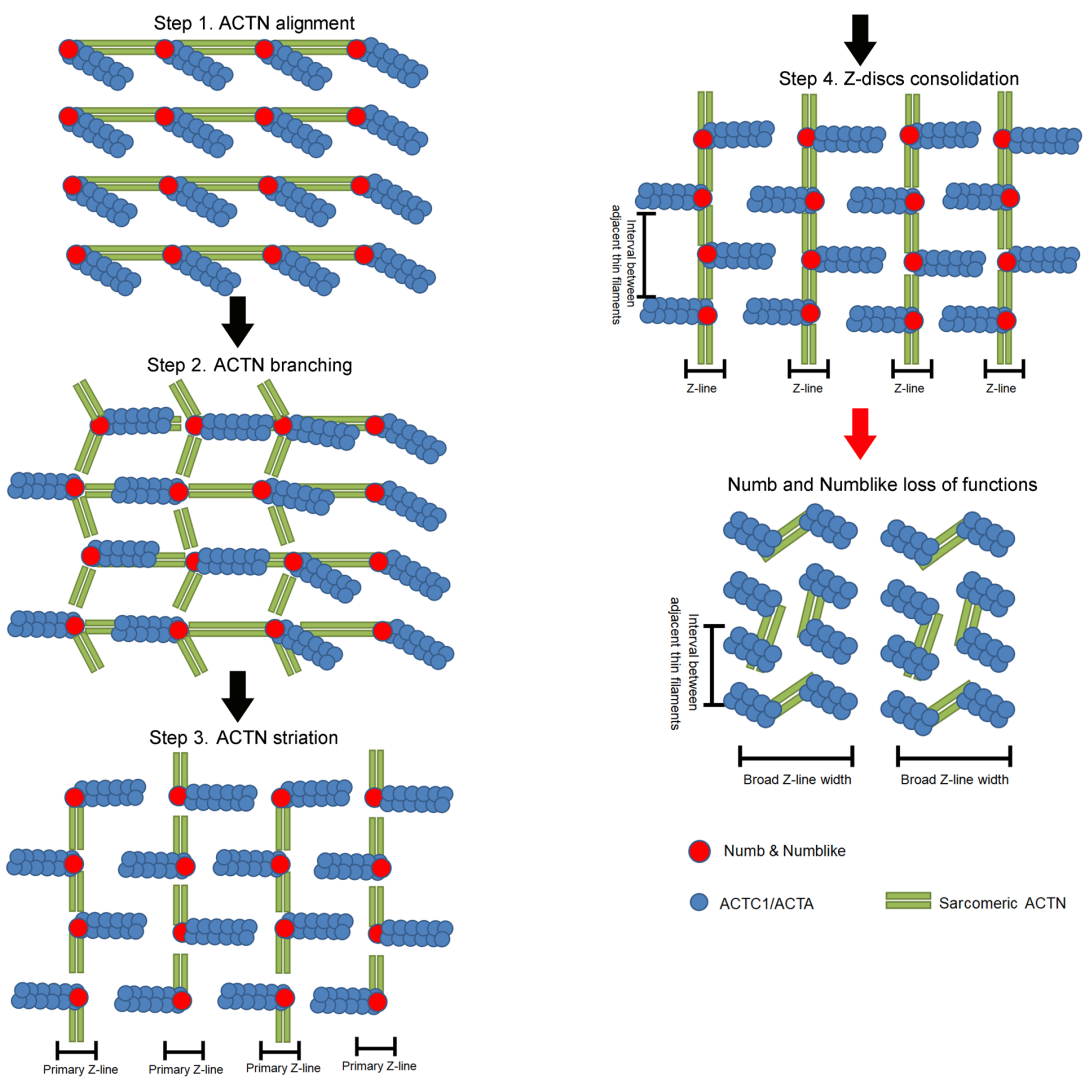
A schematic illustration of how Numb and Numblike regulate sarcomere assembly and maintenance. Numb and Numblike bind to sarcomeric α-Actin to regulate sarcomeric ACTN alignment, branching, and striation. The loss of function of Numb and Numblike compromises thin filament assembly and Z-disc consolidation.

In summary, this study identified the role of Numb and Numblike in sarcomeric thin filament formation and demonstrated the putative underlying mechanisms that regulate sarcomere assembly and maintenance in striated muscles (summarized in Figure 6). Our findings provide the molecular basis for the critical role of Numb family proteins in cardiac development. Taken together with the recent findings that Numb and Numblike modulate heart morphogenesis, we believe that these proteins might represent a potential therapeutic target for cardiac dysfunction and heart diseases that are associated with failure in assembly and maintenance of the cardiac sarcomere.

## Methods

### Animals and cell lines.

Mouse strains *Numb^fl/fl^ Numblike ^fl/fl^*, *MLC-2v-cre*, and α*-MHC-cre* were purchased from the Jackson Laboratories. *Mesp1-cre* and *CKMM-cre* mouse strains were purchased from Nanjing Laboratory Animal Center. *Numb^fl/fl^ Numblike ^fl/fl^* mice were mated to *Mesp1-cre*, *MLC-2v-cre*, α*-MHC-cre*, and *CKMM-*cre mice to generate Numb *^fl/+^* Numblike *^fl/+^* heterozygous mice. Then heterozygous mice were mated to *Numb^fl/fl^ Numblike ^fl/fl^* mice to generate *Numb* and *Numblike* DKO mice. All mouse breeding was done under specific pathogen–free conditions. We conducted all the animal experiments in accordance with the university guidelines and under the approval of the Animal Care Committees at Sichuan University. The H9C2 (CRL-1446) and C2C12 (CRL-1772) cell lines were purchased from ATCC and the AD293 (Adeno-X 293, 632271) cell line was purchased from Clontech.

### X-gal staining.

The embryos or small tissue pieces were dissected in PBS on ice and then fixed with 4% paraformaldehyde overnight at 4°C. Samples were rinsed with PBS 3 times and then incubated at 37°C for 2 to 4 hours in X-gal (Thermo Fisher Scientific, B1690) solution diluted in the X-gal Reaction Buffer (35 mM potassium ferricyanide, 2 mM MgCl_2_, 0.02% Nonidet P-40, 0.01% Na deoxycholate). After the incubation, the samples were rinsed with PBS until the solution was no longer yellow.

### Transmission electron microscopy, hematoxylin & eosin staining, and immunostaining.

Transmission electron microscopy was performed as previously described (36). Hematoxylin & eosin staining was carried out as described (37). For immunostaining, embryos or tissues were fixed with 4% paraformaldehyde overnight at 4°C and then rinsed with PBS 3 times. The samples were dehydrated in an increasing gradient of 10% to 30% sucrose/PBS and embedded in OCT (Tissue Tek). The samples were cryosectioned at 10-μm thickness, and OCT was washed out by PBS. The sections were incubated with diluted primary antibody overnight at 4°C and then with cy2- or cy3-labeled secondary antibody (Jackson Immunoresearch) for 1 hour at room temperature with PBS wash 3 times in between. The following antibodies were used for immunostaining: rabbit-anti-Numb (Cell Signaling, 2756s, 1:200 dilution), mouse anti-α-Actinin (Sigma, A7811, 1:500 dilution), mouse anti-α-Actin (Sigma, A9357, 1:500 dilution), goat anti-NEBL (Abcam,ab99420, 1:100 dilution), mouse anti-α-Tubulin (Covance, MMS-407R, 1:800 dilution), mouse anti-Desmin (Covance, MMS-454s, 1:200 dilution), and Phalloidin (Invitrogen, A12379 1:500 dilution).

### Western blot and coimmunoprecipitation.

Western blot samples were rapidly washed with ice-cold PBS 3 times and homogenized in lysis buffer (25 mM HEPES, pH 7.0, 5 mM MgCl_2_, 25 mM KCl, 0.05 mM EDTA, 0.1% NP-40, 10% glycerol). The supernatants were collected after boiling and centrifugation. Blots were then incubated with the primary antibodies: rabbit anti-Numb (Cell Signaling, 2756s, 1:1000 dilution), mouse anti-α-Actinin (Sigma, A7811, Cell Signaling, 2756s, 1:2000 dilution), mouse anti-α-Actin (Sigma, A9357, 1:2000 dilution), rabbit anti-N-wasp (Cell Signaling, 4848s, 1:1000 dilution), rabbit anti-PFN-1 (Epitomics, 3934-1, 1:1000 dilution), goat anti-NEBL (Abcam, ab99420, 1:500 dilution), mouse anti-α-Tubulin (Covance, MMS-407R, 1:2000 dilution), or rabbit anti-GAPDH (Cell Signaling, 2118s, 1:2000 dilution). After the incubation, the blots were incubated with HRP-conjugated anti-mouse IgG, HRP-conjugated anti-goat IgG, or HRP-conjugated anti-goat IgG (Jackson ImmunoResearch Laboratories, 1:2000 dilution). The chemiluminescence was detected using ECL Western blot detection kits (GE Healthcare Bio-Sciences) according to the manufacturer’s protocol.

For coimmunoprecipitation, cell or tissue lysates were rapidly washed with ice-cold PBS 3 times and then homogenized in NP-40 lysis buffer (25 mM K-HEPES, 12.5 mM MgCl_2_, pH 7.5, 250 mM KCl, 8% glycerol, 0.5% NP-40, 1 mM dithiothreitol [DTT], phosphatase and protease inhibitors cocktail). The supernatants were collected after centrifugation and incubated with the primary antibodies: rabbit anti-Numb (Cell Signaling, 2756s, 1:100 dilution), mouse anti-α-Actin (Sigma, A9357, 1:200 dilution), rabbit anti-N-wasp (Cell Signaling, 4848s, 1:100 dilution), or anti-Myc-Tag (Cell Signaling, 2276, 1:500 dilution) overnight with rotation at 4°C. Lysates were then incubated with Protein A/G beads for 2 hours with rotation at 4°C to precipitate the complexes. When the incubation time was over, the tubes were centrifuged, supernatant removed, and the beads washed in lysis buffer three times. Finally, the last supernatant was removed, and an appropriate volume of loading buffer was added. Lysates were boiled at 95°C–100°C for 5 minutes to denature the protein and separate it from the protein A/G beads, then centrifuged and run on a SDS-PAGE. Blots were incubated with the following antibodies: rabbit anti-Numb (Cell Signaling, 2756s, 1:1000 dilution), mouse anti-α-Actinin (Sigma, A7811, Cell Signaling, 2756s, 1:2000 dilution), mouse anti-α-Actin (Sigma, A9357, 1:2000 dilution), rabbit anti-N-wasp (Cell Signaling, 4848s, 1:1000 dilution), rabbit anti-PFN-1(Epitomics, 3934-1, 1:1000 dilution), goat anti-NEBL (Abcam, ab99420, 1:500 dilution), anti-Myc-Tag (Cell Signaling, 2276, 1:2000 dilution), anti-GFP-Tag (NeuroMab, 75-132, 1:2000 dilution), anti-His-Tag (applygen, C1301, 1:2000 dilution), or anti-Flag-Tag (Cell Signaling, 2368, 1:2000 dilution). After the incubation, the blots were incubated with HRP-conjugated anti-mouse IgG, HRP-conjugated anti-goat IgG, or HRP-conjugated anti-goat IgG (Jackson ImmunoResearch Laboratories, 1:2000 dilution). The chemiluminescence was detected with ECL Western blot detection kits (GE Healthcare Bio-Sciences) according to the manufacturer’s protocol.

### Electrocardiographic analysis and gastrocnemius tension measurement.

Electrocardiogram was obtained for the mouse heart by electrophysiological equipment, and electrocardiographical analysis was performed as previously described (38). For gastrocnemius tension measurement, mice were euthanized by cervical dislocation. The gastrocnemius muscle from the leg of the mouse was isolated, one posterior end of gastrocnemius was fixed flat on a board, and the anterior end was connected to electrophysiological equipment for muscle contraction detection. The applied voltage was gradually increased, and the contractility data were recorded and analyzed.

### Primary cardiomyocyte culture.

CM isolation and immunohistochemical experiments were performed as previously described (39).

### Actin polymerization assay and TIRF microscopy.

Actin Polymerization Assay was performed according to the manufacturer’s protocol (Cytoskeleton, catalog BK003). TIRF microscopy was conducted according to the previously described protocol (33).

### RT-PCR.

First-strand cDNAs were synthesized using TransScript One-step gDNA Removal and cDNA synthesis SuperMix kit (TransGen Biotech, catalog AT311-02) according to the manufacturer’s protocol. The sequences of the primers used for PCR amplifications were listed as follows: Numb, forward primer 5′-CGTCACTGCTACTTTTGATGCCAGTAGAAC-3′and reverse primer 5′-AAGAGCCTTGACGAGCAAGCTGTTCAATT-3′; Numblike, forward primer 5′-GTGAAGGGCACAGTGCCTGAGATGGAGCCT-3′ and reverse primer 5′-TTGCTGCTGCTGTTGTTGCTGCTGCTGCTG-3′; GAPDH, forward primer 5′-GTGAAGGTCGGTGTGAACGGAT-3′and reverse primer 5′-ATCACGCCACAGCTTTCCAGAG-3; Numb isoforms, PTB forward primer: 5′-ACGTAGAAGTTGATGAGTC-3′and PTB reverse primer: 5′-CACAGAACGGCCTTCACTGCT-3′.

### Vector construction, transfection, shRNA knockdown.

*Numb* and *Numblike* cDNA were cloned into pcDNA3.1-Myc (Invitrogen) or pEGFP-C1 (Clontech, catalog 6084-1) vector; truncated *Numb* and *Numblike* cDNA were cloned into pcDNA3.1-Myc (Invitrogen) vector; *ACTC1*, *ACTA*, *ACTN2*, *ACTN3*, *PFN1*, *Nebulin-SH3*, *Nebulette*, *Nebulette-SH3,* and *N-WASP* were cloned into pEGFP-C1 (Clontech, catalog 6084-1) vector, respectively. shRNAs were designed and cloned into pLKO.1 vector (Thermo Fisher Scientific) and pLVX-shRNA2 vectors (Clontech, catalog 632179).

AD293 cells were cultured in Dulbecco’s modified Eagle medium (DMEM) supplemented with 10% FBS, 2 mM l-glutamine, 1 mM sodium pyruvate, and 4.5 mg/mL glucose. Plasmid DNA was transfected using Megtran 1.0 (Origene) following the manufacturer’s protocol. After taking the fluorescence image, the transfected cells were harvested 24 hours after transfection and analyzed by immunoprecipitation or Western blotting.

To generate the *Numb* and *Numblike* DKO H9C2 cell line, pLKO.1-derived lentivirus vectors (Thermo Fisher Scientific) were used to infect H9C2 cells, and the positive cells were selected by adding puromycin (Thermo Fisher Scientific, catalog A1113802, 2 μg/m) for 2 weeks. The selected cells were then infected by pLVX-shRNA2–derived lentivirus vectors (Clontech, catalog 632179) and sorted out by flow cytometry. Rat shRNA nucleotide sequences were listed as follows: Numb shRNA1 5′-GAAGGATCATTCCGTGTCACA-3′, Numb shRNA2 5′-GCAGCAGACATTCCCTCAATA-3′, Numblike shRNA1 5′-GACAAGGCATTCTCCTACATA-3′, Numblike shRNA2 5′-GACCTTCGAGATTGAACTGTA-3′, ACTC1 shRNA 5′-GAGGCATCCTGACTCTGAAGT-3′.

### Statistics.

Statistical analyses were performed using Student’s *t* test and the results were reported as mean ± SD. Differences between groups were considered statistically significant at *P* less than 0.05.

### Study approval.

Mice were maintained and bred in the specific pathogen–free grade laboratory animal facility. All animal procedures were approved by the IACUC of the West China Second Hospital (Sichuan University) and performed in accordance with the Development and Stem Cell Research Institute Guide for the Care and Use of Laboratory Animals.

## Author contributions

BW designed the project, conducted experiments, and acquired, analyzed, and interpreted data. MY assisted with Western blot, plasmid constructions, and some animal experiments. BW and MY wrote the manuscript and explained the data. SL conducted or assisted in conducting all the animal experiments.

## Supplementary Material

Supplemental data

## Version 1 02/01/2022

Electronic publication
