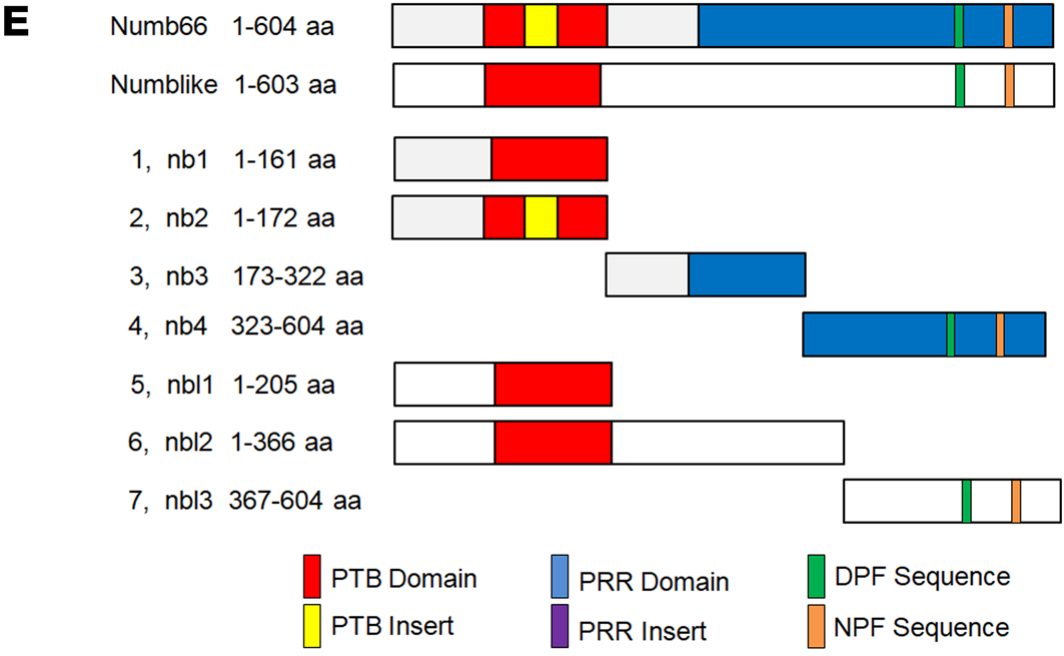# Numb and Numblike regulate sarcomere assembly and maintenance

**DOI:** 10.1172/JCI162883

**Published:** 2022-07-15

**Authors:** Baolei Wang, Min Yang, Shujuan Li

Original citation: *J Clin Invest*. 2022;132(3):e139420. https://doi.org/10.1172/JCI139420

Citation for this corrigendum: *J Clin Invest*. 2022;132(14):e162883. https://doi.org/10.1172/JCI162883

Following the publication of this article, the authors noted several errors that require correction. All observations ascribed to day p60 were actually made at p60.5. Furthermore, in Figure 3E, construct 6 was inaccurately depicted. The correct panel is shown below.

The description of Figure 3E in the Results section has also been corrected, as below:

The results indicate that Numb (173–322 aa) and Numblike (1–366 aa) were the dominant residue sequences that interacted with ACTC1 and that Numblike (1–205 aa) and Numblike (367–604 aa) had negligible functional interactions with ACTC1 (Figure 3F). After blasting the Numb (173–322 aa) and Numblike (1– 366 aa) amino acid sequences in the NCBI database, we found that these 2 sequences were highly conserved, indicating the potentially critical role of these conservative domains in α-Actin binding (Supplemental Figure 9A).

In the Discussion section, a corrected sentence has been included, as below:

Since the expression of MLC-2v is restricted within the ventricular chamber throughout mouse embryonic development, this mouse line serves as a great platform to study how Numb and Numblike affect sarcomere assembly in ventricular CMs at the early stage (19, 26).

The text and Figure 3E have been updated in the HTML version and PDF with the correct information.

The authors regret the errors.

## Figures and Tables

**Figure:**