# Supplementary material for: Numb and Numblike regulate sarcomere assembly and maintenance
Source: J Clin Invest. 2022 Feb 1;132(3):e139420. doi: 10.1172/JCI139420 (PMC8803338; doi:10.1172/JCI139420)

Supplementary data 1

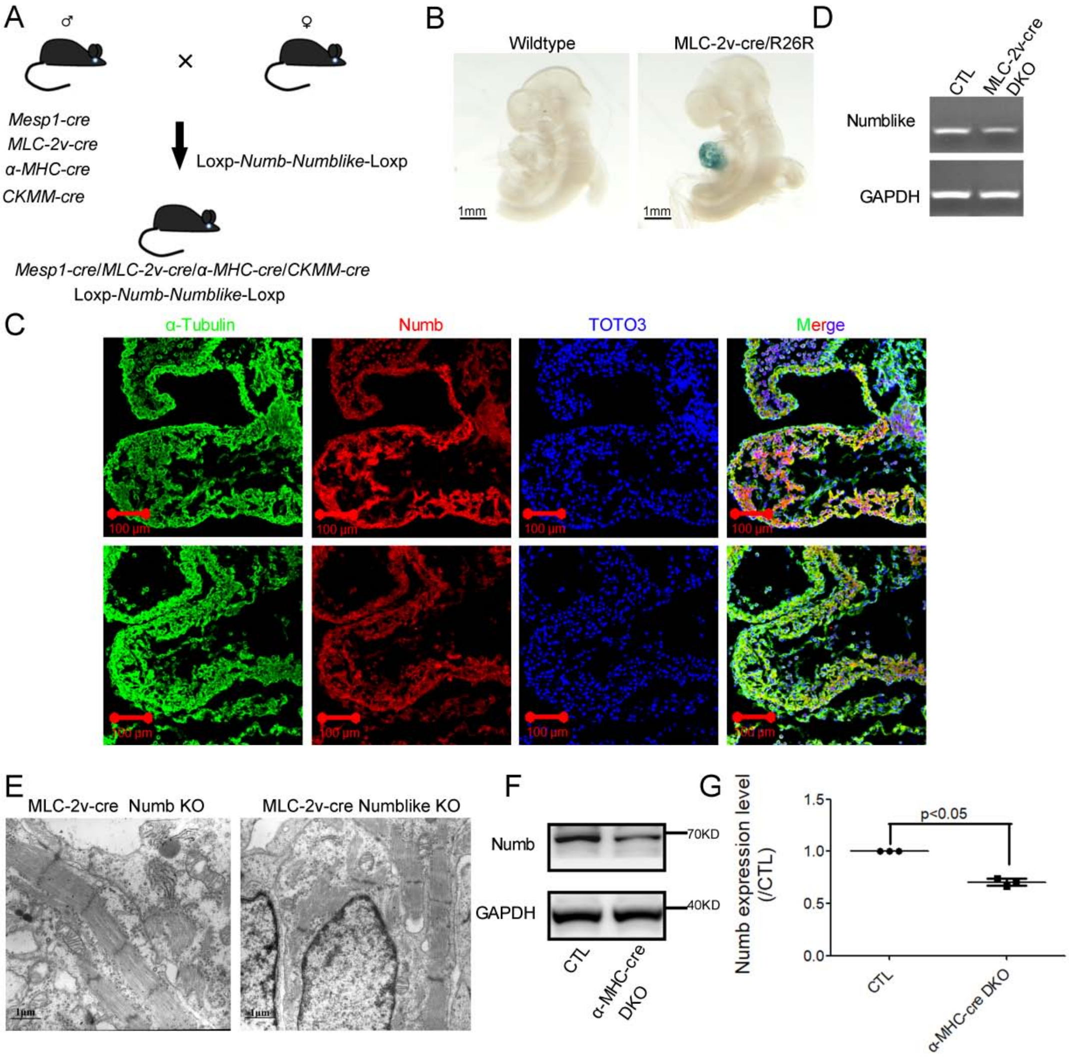

Supplementary Tab. 1 Cre type and expression cells

Cre type	Expression cells
Mesp1-cre	Cardiac progenitor cells
MLC-2v-cre	Ventricle cardiomyocytes
α-MHC-cre	Adult cardiomyocytes
CKMM-cre	Skeletal muscle cells & cardiomyocytes

Supplementary Tab. 2 Sarcomere changes in *Numb* and *Numblike* DKO mice

Cre	Genotype	Z-disc width (/CTL)	Sarcomere length (/CTL)	Interval between the adjacent thin filaments (/CTL)
MLC-2v-cre (CM)	*Numb* KO	0.97±0.51	0.96±0.06	1.1±0.02
	Numblike KO	1.05±0.32	1.02±0.14	1.08±0.02
	DKO	1.96±0.99***	0.80±0.1***	0.50±0.01***
α-MHC-cre (CM)	DKO	2.73±0.85***	0.74±0.07***	0.48±0.10***
CKMM-cre (SKM)	DKO	7.90±2.75***	0.69±0.02***	0.51±0.15*
CKMM-cre (CM)	DKO	2.47±1.03*	0.85±0.05**	0.51±0.12**

Each index of *Numb* and *Numblike* DKO were compared with CTL, n=5, Student's t test, *$P <$ 0.05,**P<0.01, ***P<0.001.

Supplementary data 2

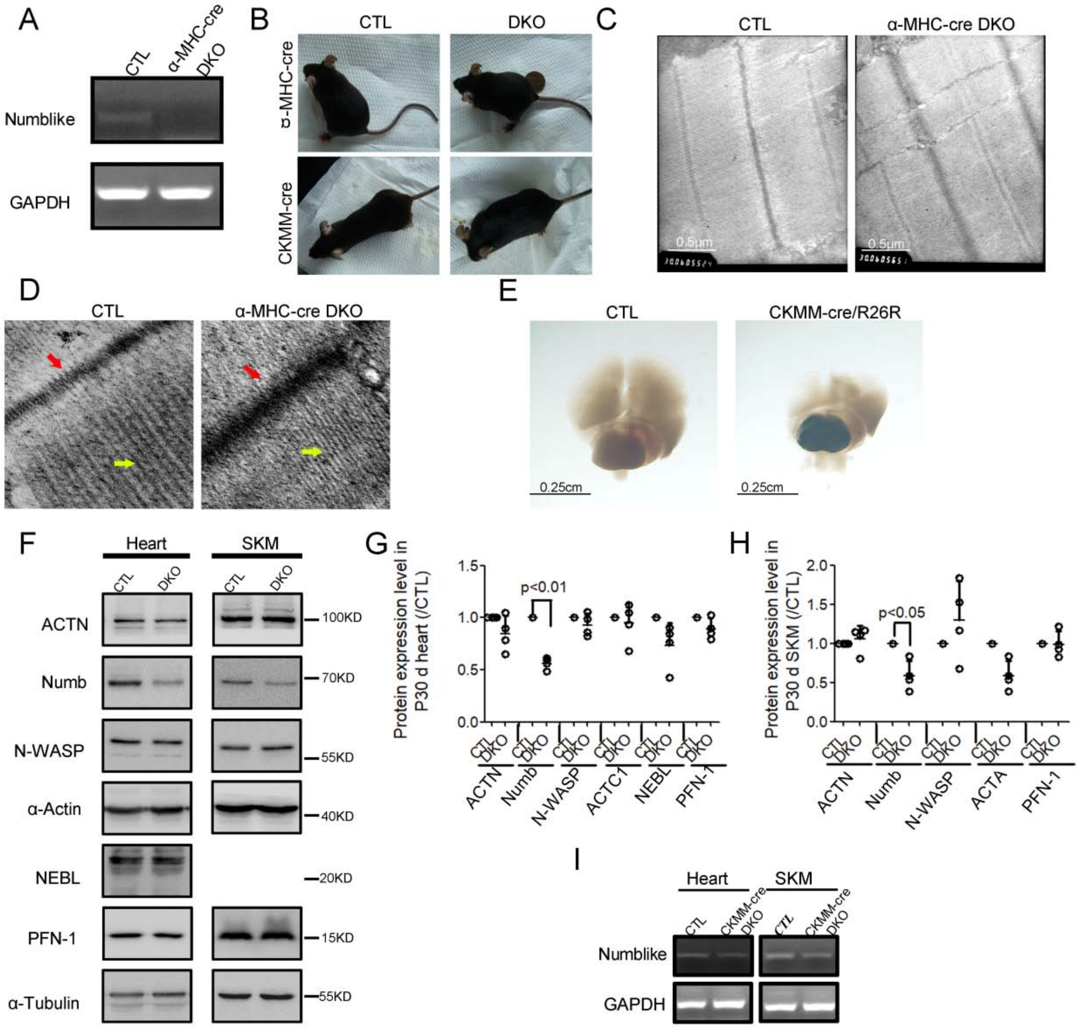

Supplementary data 3

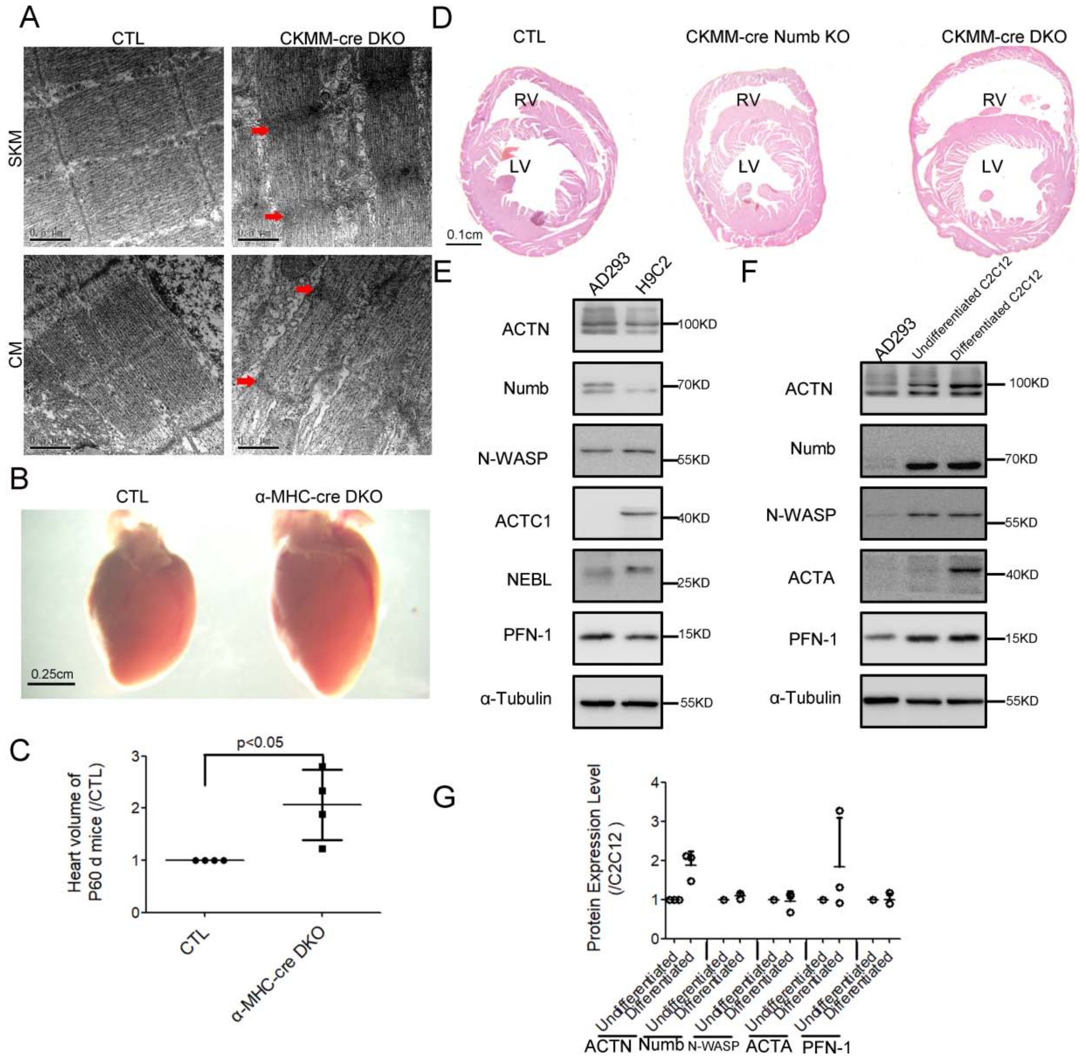

Supplementary data 4

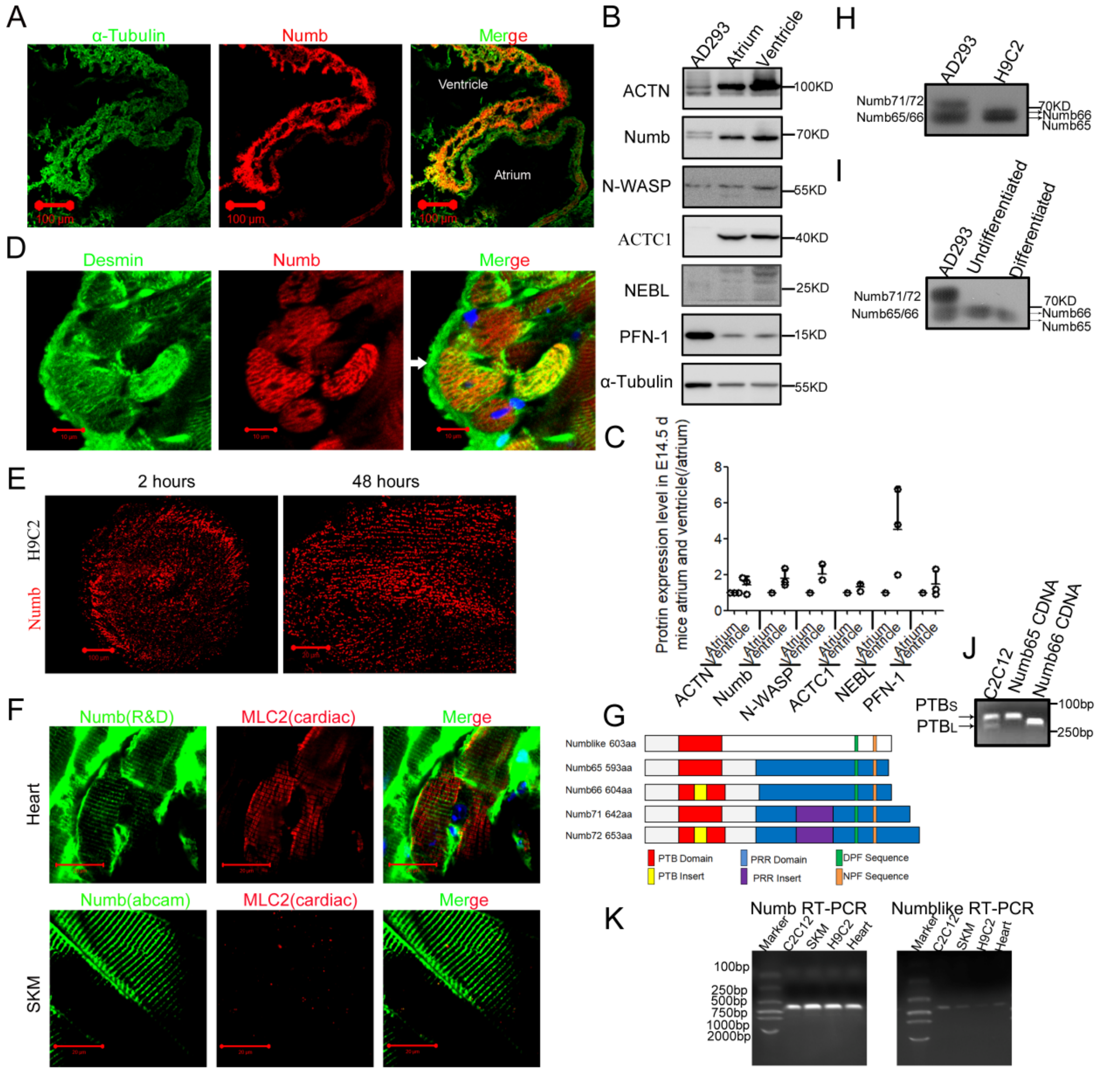

Supplementary data 5

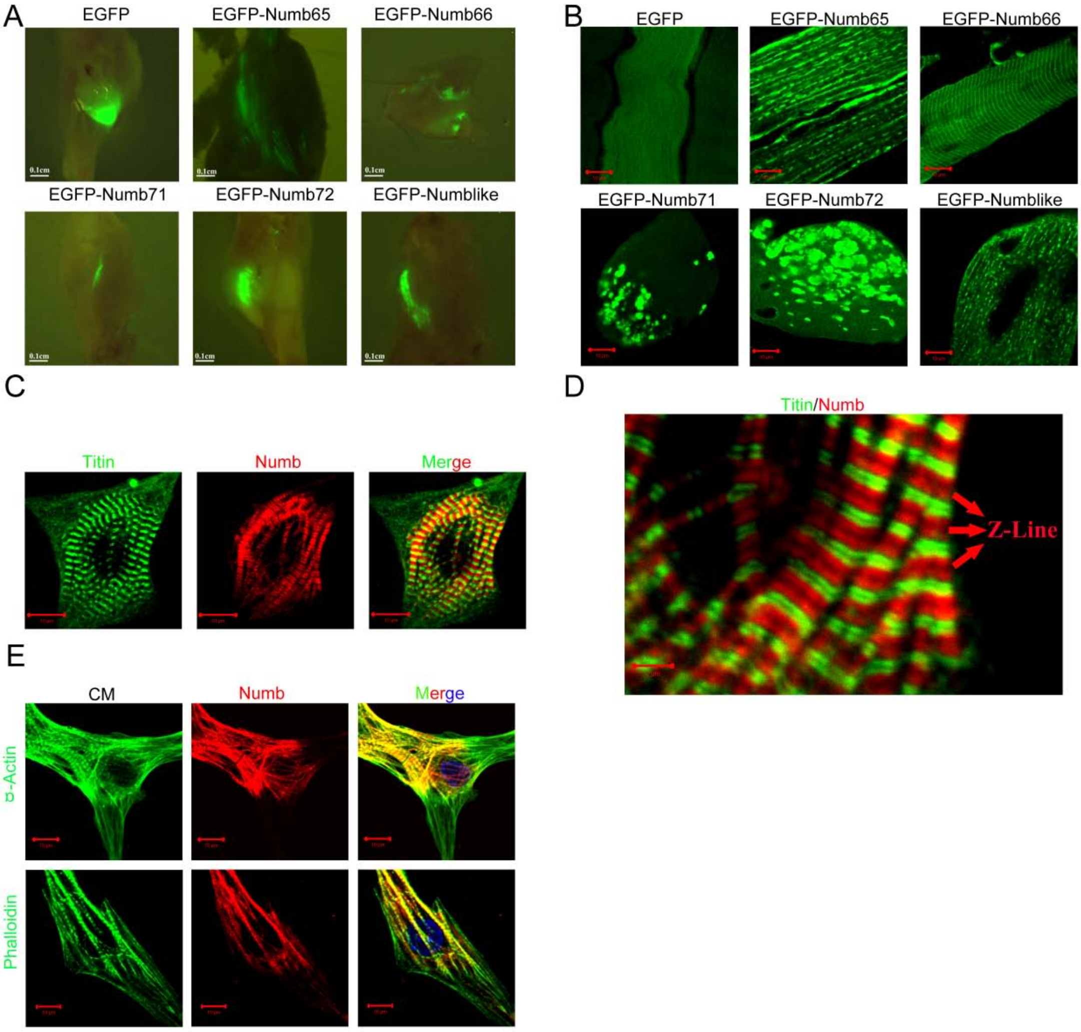

Supplementary data 6

A

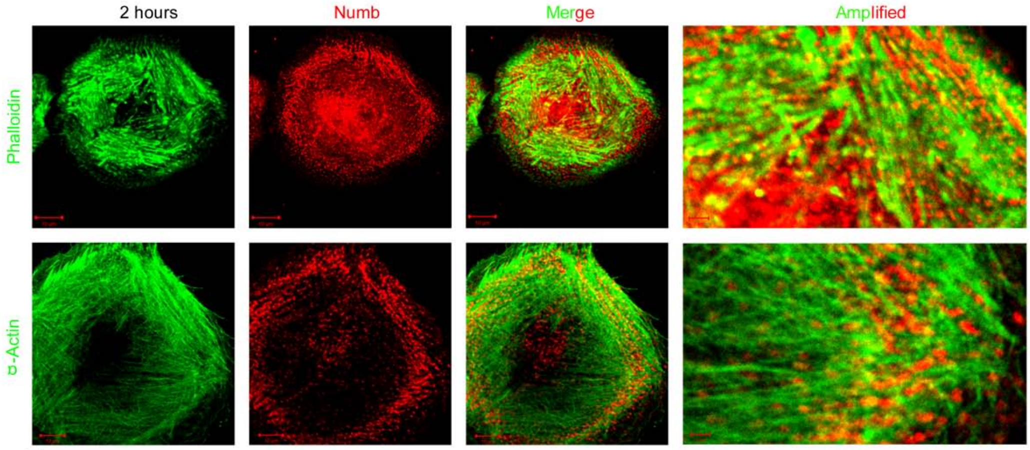

B

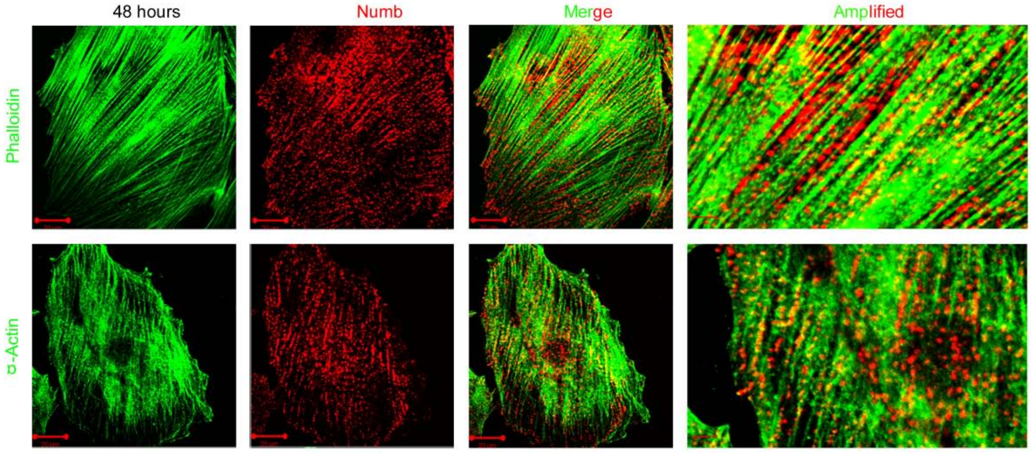

Supplementary data 7

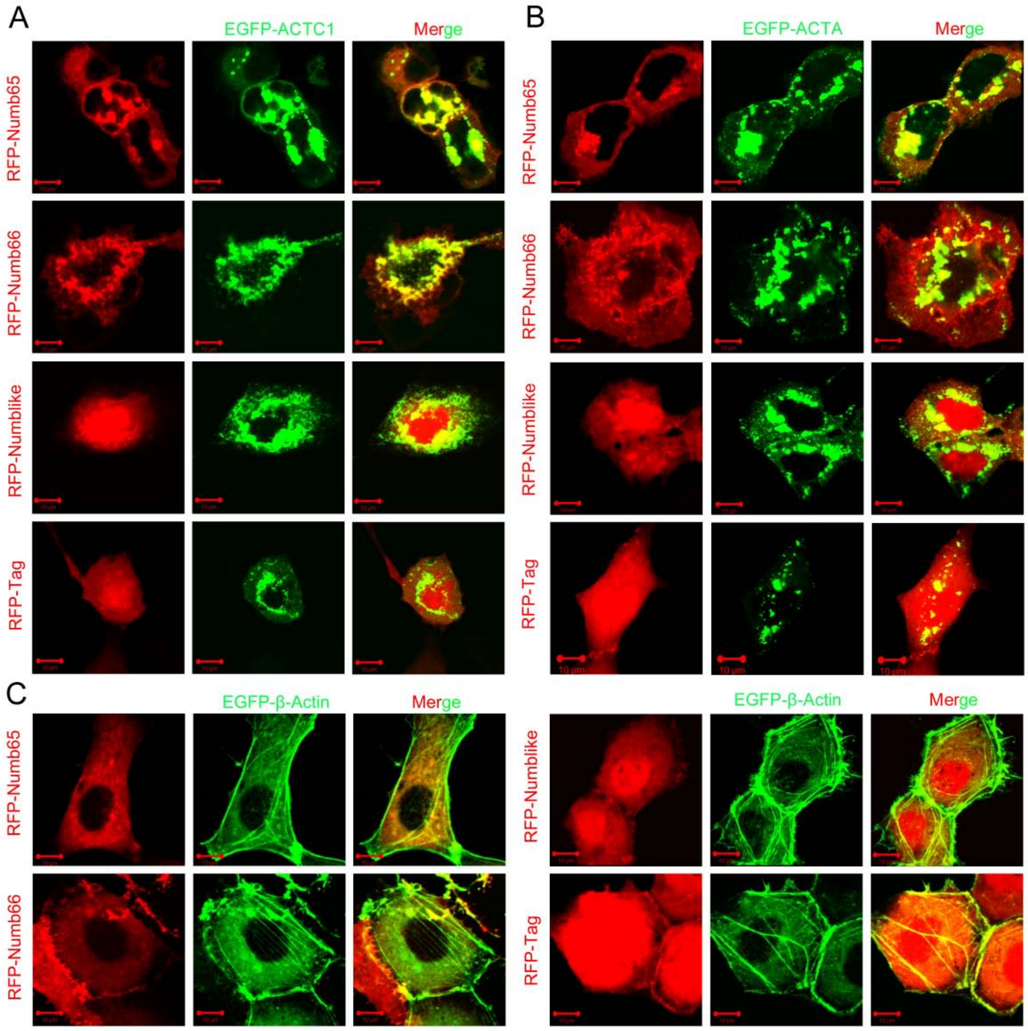

Supplementary data 8

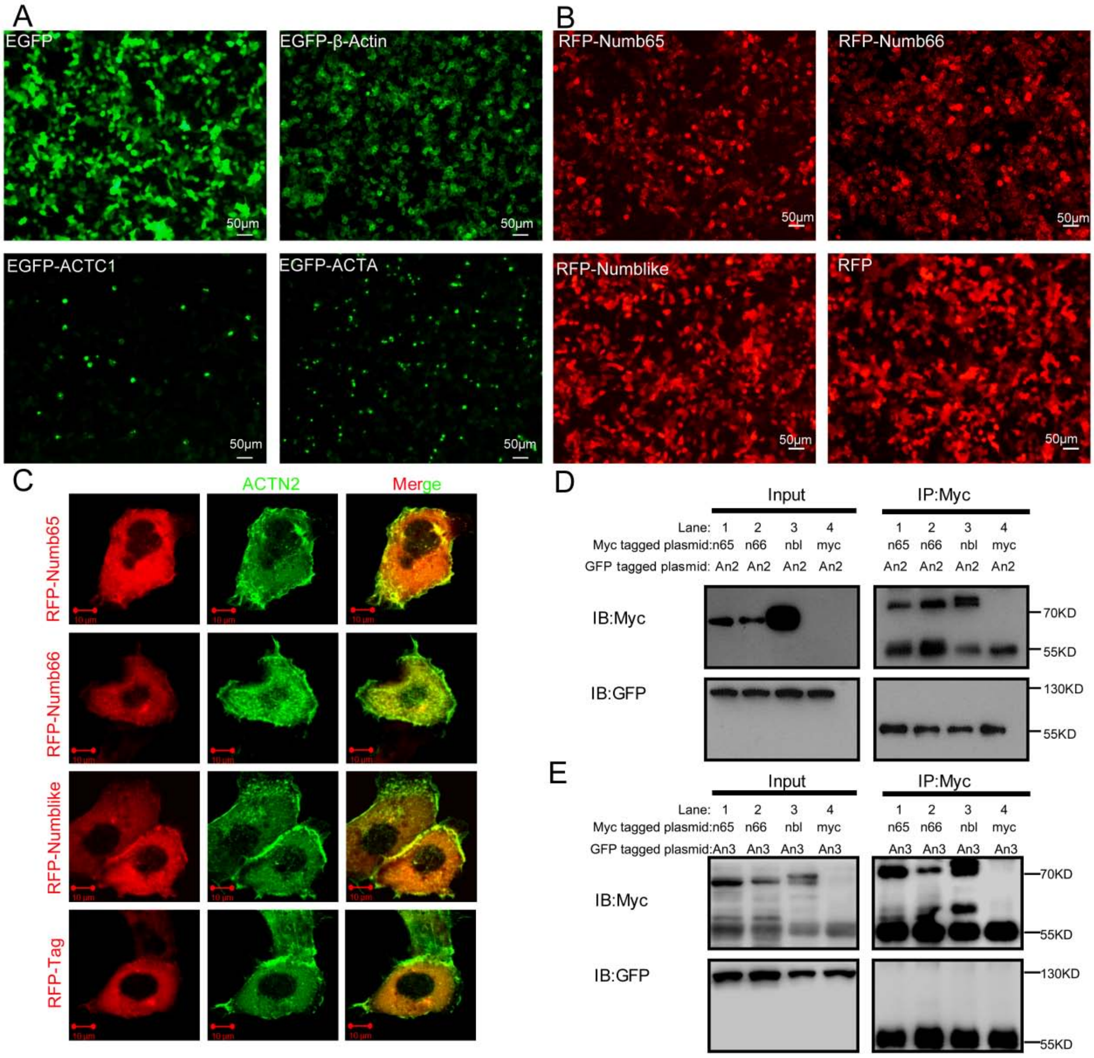

Supplementary data 9

A

Query*: *Numb residue

Subject*: *Numblike residue

```
Query 1   DASRTTFTREGSFRVTTATEQAEREEIMKQLQDAKKAETDKTAVGPSVAPGNTAPSPSSP  60
          DASRT+F REGSFR++   AERE  K+   K+A        PG+ +P+P++
Sbjct 9   DASRTSFAREGSFRLSGGGRPAEREAGDKK----KEAAAAPAVAPGPAQPGHVSPTPAT-  63

Query 61  TSPTPDGTASSEM---NNPHAIPRRHAPIEQLARQGSFRGFPALSQKMSPFKRQLSLRIN  117
          TSP  G A + +     AIPRRHAP+EQL RQGSFRGFPALSQK SPFKRQLSLR+N
Sbjct 64  TSPGEKGEAGTPVAAGTTAAAIPRRHAPLEQLVRQGSFRGFPALSQKNSPFKRQLSLRLN  123

Query 118 ELPSTMQRKTDFPIKNTVPEVE----GEAESISSLCSQ  151
          ELPST+QR+TDF +K TVPE+E   G+++ I++LC+Q
Sbjct 124 ELPSTLQRRTDFQVKGTVPEMEPPGTGDSDGINALCTQ  161
```

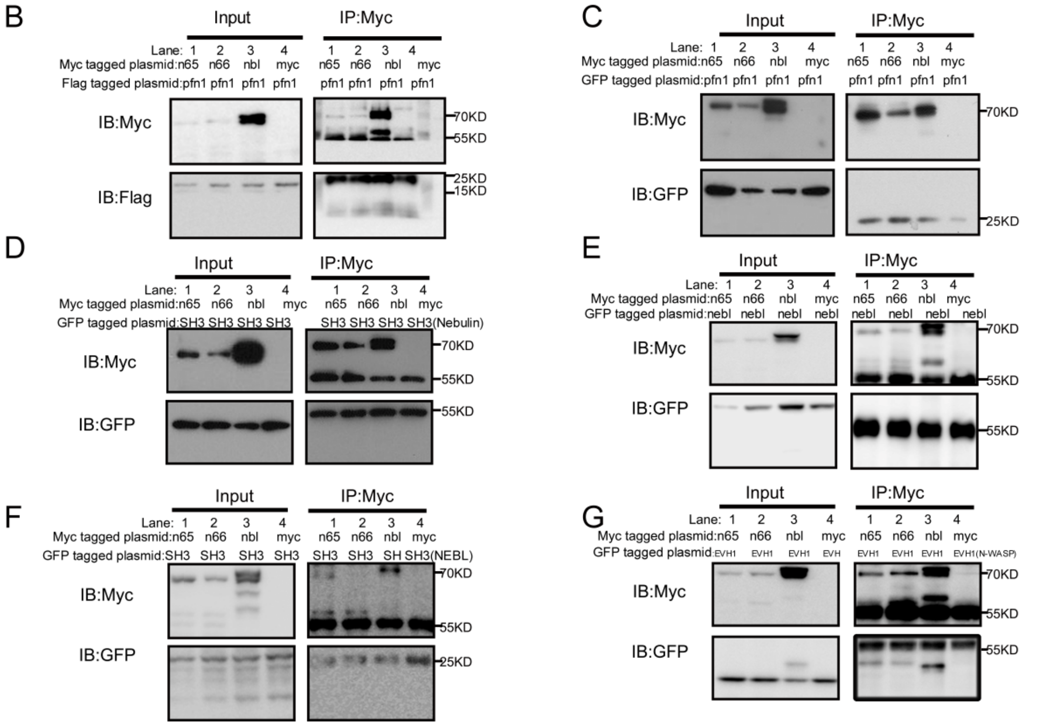

Supplementary data 10

A

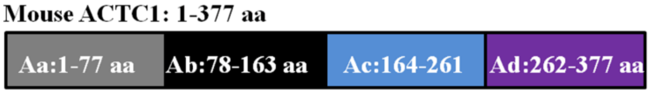

B

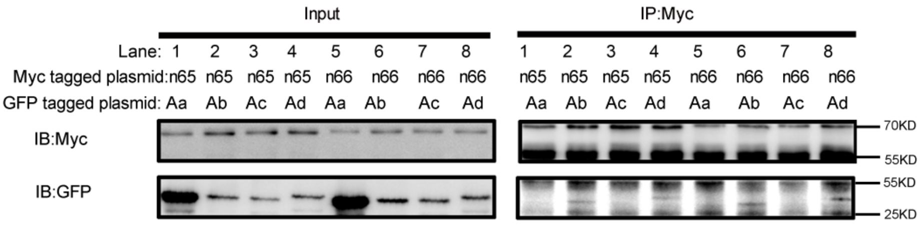

C

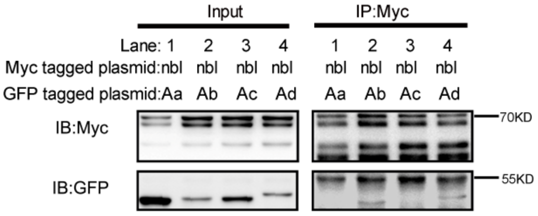

D

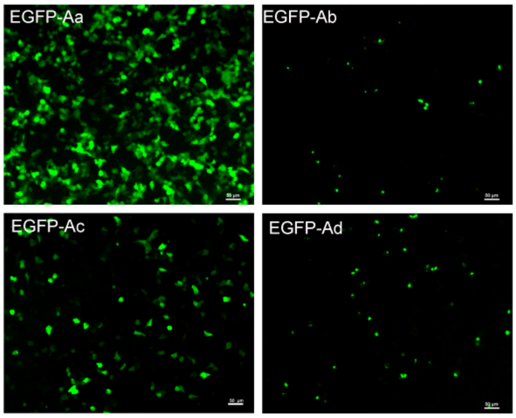

Supplementary data 11

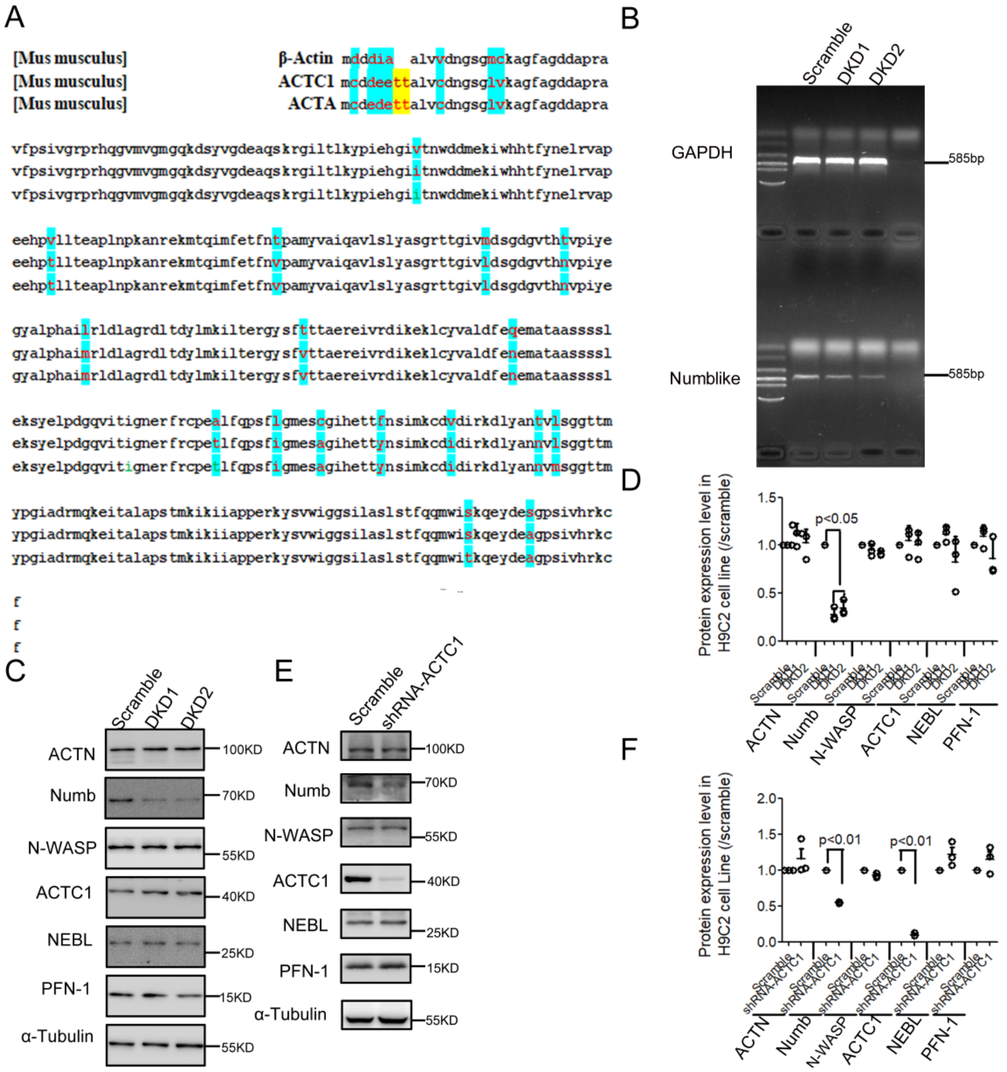

Supplementary data 12

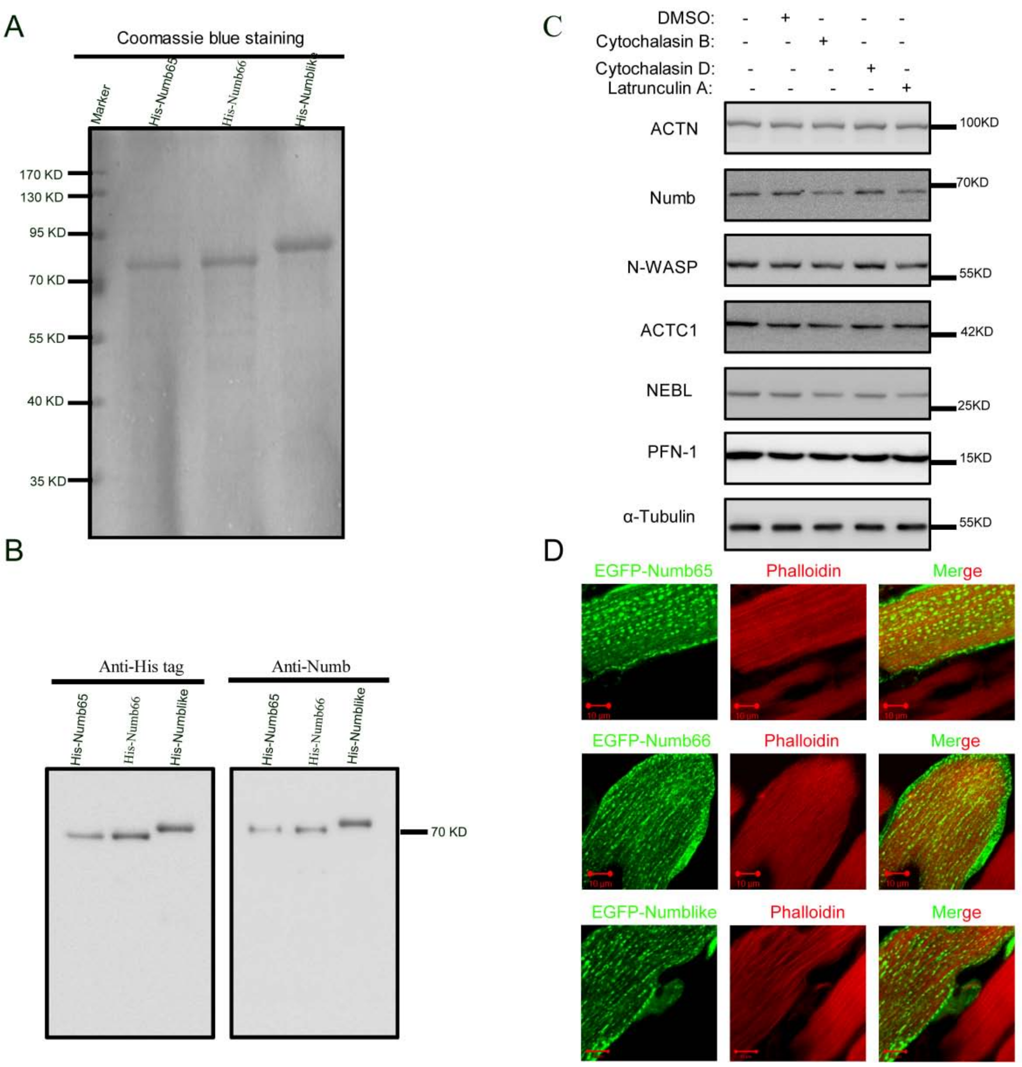

Supplementary data 13

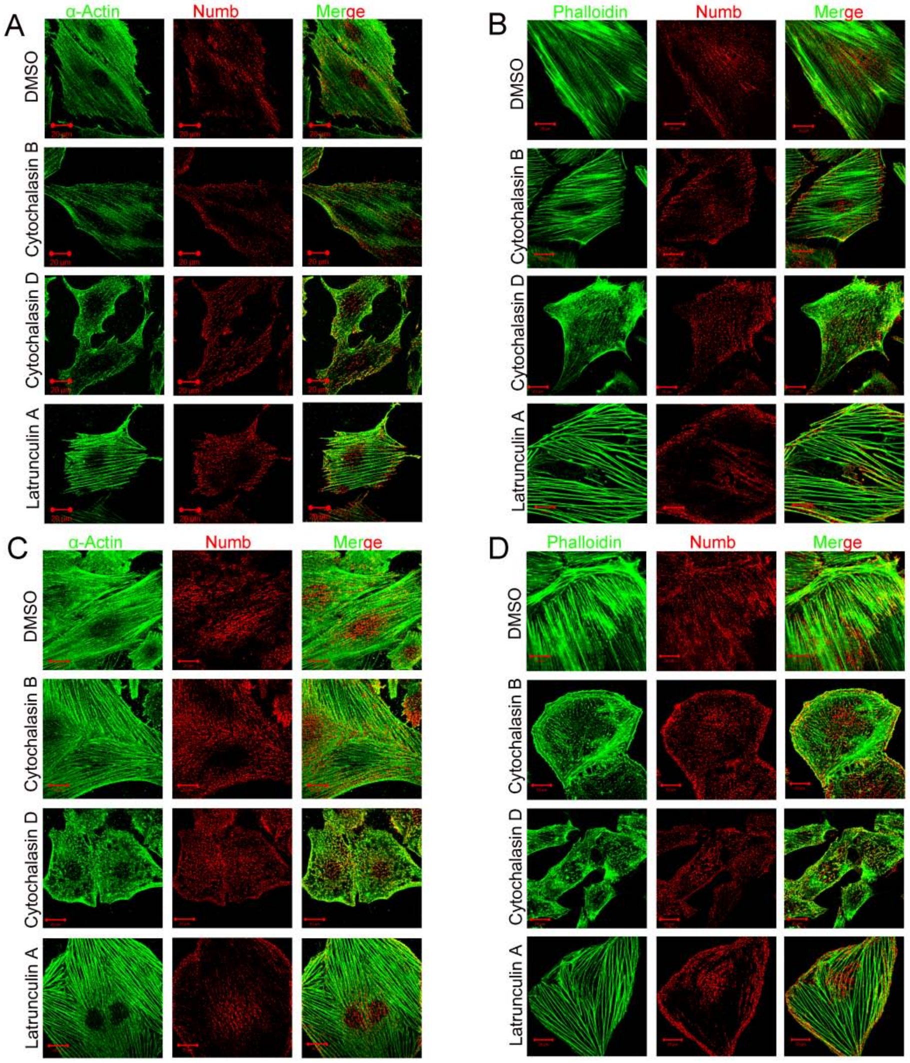

Supplement: Supplemental data [file jci-132-139420-s039.pdf]
